# Transcriptomic, Proteomic, and Bioelectrochemical Characterization of an Exoelectrogen *Geobacter soli* Grown With Different Electron Acceptors

**DOI:** 10.3389/fmicb.2018.01075

**Published:** 2018-06-15

**Authors:** Xixi Cai, Lingyan Huang, Guiqin Yang, Zhen Yu, Junlin Wen, Shungui Zhou

**Affiliations:** ^1^Fujian Provincial Key Laboratory of Soil Environmental Health and Regulation, College of Resources and Environment, Fujian Agriculture and Forestry University, Fuzhou, China; ^2^Guangdong Key Laboratory of Integrated Agro-Environmental Pollution Control and Management, Guangdong Institute of Eco-Environmental Science and Technology, Guangzhou, China

**Keywords:** comparative transcriptomics, comparative proteomics, bioelectrochemistry, *Geobacter soli*, extracellular electron transfer

## Abstract

The ability of *Geobacter* species to transfer electrons outside cells enables them to play an important role in biogeochemical and bioenergy processes. Our knowledge of the extracellular electron transfer (EET) process in the genus *Geobacter* is mainly from the study of *G. sulfurreducens*, and in order to fully investigate the EET mechanisms in the genus *Geobacter*, other *Geobacter* species should also be considered. This study focused on the EET of *Geobacter soli* GSS01, which exhibited a capability of reducing insoluble Fe(III) oxides and generating electrical current comparable with *G. sulfurreducens* PCA. Electrochemical characterization, including cyclic voltammetry, differential pulse voltammetry, and electrochemical *in situ* FTIR spectra, revealed that different redox proteins contributed to the electrochemical behaviors of *G. soli* and *G. sulfurreducens*. Based on comparative transcriptomic and proteomic analyses, OmcS was the most upregulated protein in both *G. soli* and *G. sulfurreducens* cells grown with insoluble Fe(III) oxides vs. soluble electron acceptor. However, the proteins including OmcE and PilA that were previously reported as being important for EET in *G. sulfurreducens* were downregulated or unchanged in *G. soli* cells grown with insoluble electron acceptors vs. soluble electron acceptor, and many proteins that were upregulated in *G. soli* cells grown with insoluble electron acceptors vs. soluble electron acceptor, such as OmcN, are not important for EET in *G. sulfurreducens*. We also identified 30 differentially expressed small RNAs (sRNAs) in *G. soli* cells grown with different acceptors. Taken together, these findings help to understand the versatile EET mechanisms that exist in the genus *Geobacter* and point to the possibility of sRNA in modulating EET gene expression.

## Introduction

The *Geobacter* genus has a remarkable respiratory versatility that includes the dissimilatory reduction of insoluble metal oxides in natural habitats and electron transfer to electrode surfaces from which electricity can be harvested (Mahadevan et al., 2006; Lovley, 2008). In this process, electron transfer from the inner membrane quinone/quinol pool through the periplasm and outer membrane to exterior insoluble electron acceptors, termed extracellular electron transport (EET), is an intriguing aspect of microbial respiration (Shi et al., 2007; Snider et al., 2012).

*Geobacter sulfurreducens* is a well-studied representative of the *Geobacter* genus, which plays a critical role in organic matter oxidation coupled with Fe(III) oxide reduction (Caccavo et al., 1994; Speers and Reguera, 2011). Current evidence from studies with *G. sulfurreducens* has revealed that EET occurs through the redox proteins, such as *c*-type cytochromes (*c*-Cyts) and multicopper proteins (Nevin et al., 2009; Holmes et al., 2008; Kumar et al., 2017), or through electrically conductive pili (known as nanowires) (Reguera et al., 2005). Interplay of these redox proteins and nanowires forms multiple electron conduits in the genus *Geobacter* to support diverse electron transfer processes, and distinctive proteins are involved in the EET process when cells are grown with different electron acceptors (Nevin et al., 2009; Kavanagh et al., 2016). For example, *c*-Cyts PgcA can facilitate electron transfer to Fe(III) oxides but not electrodes (Zacharoff et al., 2017).

In addition, the EET pathways may differ between *Geobacter* species (Butler et al., 2010; Merkley et al., 2015). For example, comparative transcriptomics and gene deletion study has reported that, several homologous *c*-Cyts that are important for Fe(III) oxide reduction in *G. metallireducens* are not important for Fe(III) oxide reduction in *G. sulfurreducens* (Smith et al., 2013). Therefore, to fully investigate EET mechanisms in the genus *Geobacter*, other *Geobacter* species in addition to *G. sulfurreducens* should be considered.

*Geobacter soli* GSS01, one of few *Geobacter* species isolated from soil, has many environmentally significant physiological properties that are not found in *G. sulfurreducens*, such as the ability to anaerobically oxidize aromatic compounds including phenol, benzoate, and benzaldehyde (Zhou et al., 2014). *G. soli* is also capable of catalyzing both anodic and cathodic reactions in bioelectrochemical systems (Yang et al., 2017). Genome analysis has revealed that *G. soli* GSS01 contains 76 *c*-Cyts-encoding genes (Yang et al., 2015), which are much fewer than *G. sulfurreducens* PCA with 111 *c*-Cyts-encoding genes (Methé et al., 2003). All the *c*-Cyts-encoding genes in strain GSS01, with the exception of *norB* and *norC*, have homologs in strain PCA.

Here, the EET ability of *G. soli* and *G. sulfurreducens*, including electron transfer to Fe(III) oxides and electrode, was compared, and electrochemical characteristics of *G. soli* were assessed by cyclic voltammetry (CV), differential pulse voltammetry (DPV), and electrochemical *in situ* FTIR spectra. The essential components for EET in *G. soli* were discovered by performing comparative transcriptomics and proteomics in combination with protein localization prediction.

## Materials and Methods

### Bacterial Strains and Culture Conditions

*Geobacter soli* GSS01 was previously isolated in our laboratory (Zhou et al., 2014). *G. sulfurreducens* PCA (DSM 12127) was obtained from the German Collection of Microorganisms and Cell Cultures. Both strains were cultured under anaerobic conditions (N_2_:CO_2_, 80:20%) in freshwater medium (Bond and Lovley, 2003) containing acetate (16 mM) and Fe(III)-citrate (FC; 56 mM) as the electron donor and acceptor, respectively.

### Fe(III) Oxides Reduction

Four common insoluble Fe(III) oxides, ferrihydrite (FH), goethite (α-FeOOH), lepidocrocite (γ-FeOOH), and hematite (α-Fe_2_O_3_), were synthesized as previously reported (Yen et al., 2002; Mccormick and Adriaens, 2004; Borer et al., 2007). For the Fe(III) oxide reduction assay, 16 mM acetate and 50 mM Fe(III) oxides were utilized as the electron donor and acceptor, respectively. Cell growth phases were determined by measuring Fe(II) concentration. The extractable Fe(II) that formed during the Fe(III) oxide reduction was extracted with 0.5 M HCl and quantified colorimetrically using 1,10-phenanthroline (Wu et al., 2010). For each treatment, three replicates were used.

### Electrochemical Characterization

A single-chamber, three-electrode system with a volume of 7 ml was constructed using indium tin oxide electrode (ITO) (1.8 cm^2^ surface area) as the working electrode (Zhuhai Kaivo Optoelectronic Technology Co., Ltd, China), an Ag/AgCl electrode as the reference electrode (CH Instruments Inc., China), and Ti wire as the counter electrode (Baoji Eastsun Titanium Industry, Co., Ltd, China) (Supplementary Figure S1). Log-phase cells of strain GSS01 or PCA [cell density reached A_600_ of ca. 0.2; 5% (v/v) inocula] were inoculated into the chamber which contained 6 ml of freshwater medium supplemented with 16 mM acetate as the electron donor. The working electrode was poised at 0.3 V using CHI1000C electrochemical station (CH Instruments Inc., China). All potentials in this study were determined relative to the Ag/AgCl (saturated KCl) electrode, and the potential of this electrode with respect to standard hydrogen electrode is +0.197 V. The electrical current density was normalized with the anode area. The Coulombic efficiency which represented the percentage of substrate converting to electrical energy was calculated according to the method previously reported (Liu et al., 2005).

The CV, DPV, and electrochemical impedance spectroscopy (EIS) were carried out on a CHI660E electrochemical station (CH Instruments Inc., China). The parameters for the CV and DPV were as follows: CV: scan rate, 1 mV/s; *E_i_* = -0.6 V and *E_f_* = 0.3 V; DPV: *E_i_* = -0.8 V and *E_f_* = 0.2 V; pulse height, 50 mV; pulse width, 300 ms; step height, 2 mV; step time, 500 ms; scan rate, 4 mV/s; accumulation time, 5 s. The EIS was conducted at a set potential equal to the anode operating potential corresponding to the maximum electrical current density, over a frequency range of 100 kHz to 0.1 Hz, with a sinusoidal perturbation of 5 mV amplitude, and was analyzed using ZSimDemo (version 3.20) as previously described (Yang et al., 2017).

The log cells of strain GSS01 and PCA grown with FC (soluble electron acceptor) or ITO electrode (insoluble electron acceptor) were collected for electrochemical *in situ* FTIR spectra analysis, which was performed as previously described (You et al., 2015) with the following modification: the reference potential (*E_R_*) was set at -0.8 V, and sample potentials (*E_s_*) were set at potentials -0.7 to 0.3 V with an interval of 0.2 V. All the electrochemical tests were conducted in triplicates.

### Comparative Transcriptomics and Proteomics

#### Sample Preparation for Transcriptomic and Proteomic Analyses

Mid exponential phase cells of *G. soli* grown with FC (56 mM) as an electron acceptor were harvested with centrifugation at 5000 rpm for 10 min at 4°C, washed with freshwater medium twice, resuspended in freshwater medium to an A_600_ of ca. 0.3, and used as the seed culture for inoculation [5% (v/v) inocula]. To prepare samples for transcriptomic and proteomic analyses, *G. soli* was grown with acetate (16 mM) as an electron donor and FC (56 mM), FH (100 mM) or ITO electrode (poised at 0.3 V) as an electron acceptor. The FC- and FH-samples were cells at the late exponential phase (approximately 40 and 30 mM Fe(II) were produced, respectively), and the ITO-sample was prepared when the electrical current reached the maximum value at the second cycle. In order to free cells from the FH, the FH was dissolved with oxalate solution (ammonium oxalate, 28 g/L; oxalic acid, 15 g/L) according to previous studies (Lovley and Phillips, 1988; Ding et al., 2008; Leang et al., 2010). The collected cells were washed with 50 mM Tris-HCl (pH 7.8) twice, quick-frozen by liquid nitrogen, and stored at -80°C before further use. All samples were prepared under anaerobic conditions. The FC-sample was used as control and processed with two replicates for proteomics and three replicates for transcriptomics, and the FH- and ITO-samples were investigated in triplicate for both transcriptomic and proteomic analyses.

### RNA Extraction, Sequencing, and Data Analysis

Total RNA was extracted using an RNAiso Plus kit (Takara) following the manufacturer’s instructions. The integrity of RNA was verified by performing RNase free agarose gel electrophoresis, and RNA concentration was measured using a Qubit^®^ RNA Assay Kit in a Qubit^®^ 2.0 Fluorometer (Life Technologies, CA, United States). Ten microgram quantities of RNA were ribodepleted with a Ribo-Zero kit. A cDNA library was generated using a NEBNext^®^ Ultra^TM^ Directional RNA Library Prep Kit for Illumina^®^ (NEB, United States) and sequenced on an Illumina Hiseq 2000 platform; paired-end reads (100-bp length) were generated. Before assembly, adapter reads and low-quality reads containing over 50% of bases with quality scores of 5 or lower and/or over 10% unknown bases (N bases) were removed from each data set to obtain more reliable results. The raw transcriptional data have been deposited into the Sequence Read Archive (SRA) database with an accession number of SRP135234.

Sequencing reads were remapped to the reference genome using Bowtie2-2.2.3 (Langmead and Salzberg, 2012). For each gene, the expression level was measured as Fragments Per Kilobase of transcript sequence per Millions base pairs sequenced (FPKM). The differential expression analysis of two groups was performed using the DESeq R package 1.18.0 (Anders and Huber, 2010). The resulting *P*-values were adjusted using the Benjamini and Hochberg’s approaches in order to control the false discovery rate. Genes with an adjusted *P*-value ≤ 0.05 were designated as differentially expressed. RockHopper, a software designed for small RNA (sRNA) and transcriptome analysis of bacterial RNA-seq data, was used for the detection of novel small transcripts and the differential expression analyses (Dequivre et al., 2015).

#### Protein Extraction, Digestion, and Desalting

The prepared samples were dissolved in 200 μl of triethyl ammonium bicarbonate (TEAB) dissolution buffer, broken by ultrasonic waves for 15 min, and centrifuged at 12000 rpm for 20 min. The supernatant subsided by adding fourfold volumes of cold acetone containing 10 mM Dithiothreitol (DTT) for 2 h. After centrifugation at 12000 rpm for 20 min at 4°C, the precipitates were collected and mixed with 800 μl of cold acetone to break protein disulfide bonds. The samples were centrifuged again at 12000 rpm for 20 min at 4°C and dried, and the precipitates were collected, dissolved in 100 μl of TEAB dissolution buffer for later use.

Total protein concentration was measured using the Bradford method (Bradford, 1976). For each sample, 100 μg of protein was dissolved in 100 μl TEAB dissolution buffer, and then diluted with 500 μl of NH_4_HCO_3_ solution (50 mM). After it was reduced and alkylated, 2 μg of trypsin was added and then incubated overnight at 37°C for protein digestion. After protein digestion, an equal volume of 0.1% formic acid (FA) was added for acidize. Peptides were purified on a Strata –X C18 pillar which was first activated with methanol and then balanced by adding 1 ml of 0.1% FA for three times, washed with 0.1% FA+5% acetonitrile (ACN) twice, and eluted with 1 ml of 0.1% FA+80% ACN. Eluted peptides were dried with a vacuum concentration meter. The dried peptide powder was re-dissolved in 20 μl of 0.5 M TEAB for peptide labeling.

#### iTRAQ Labeling and Identification of Protein by LC-MS/MS

The prepared samples were labeled with an iTRAQ Reagent-8 plex Multiplex Kit (AB Sciex U.K. Limited) according to the manufacturer’s instructions (Wuhan Genecreate Biological Engineering Co., Ltd). Eight samples were iTRAQ labeled: 113- and 114-iTRAQ tags for FC-samples, 115-, 116-, and 117-iTRAQ tags for FH-samples, and 118-, 119-, and 121-iTRAQ tags for ITO-samples. All labeled samples were mixed in equal amounts and then fractionated using a high-performance liquid chromatography (HPLC) system (Thermo DINOEX Ultimate 3000 BioRS) containing a Durashell C18 column (5 μm, 100 Å, 4.6 i.d. × 250 mm). At last, 12 fractions were collected: 115 vs. 113, 115 vs. 114, 116 vs. 113, 116 vs. 114, 117 vs. 113, 117 vs. 114, 118 vs. 113, 118 vs. 114, 119 vs. 113, 119 vs. 114, 121 vs. 113, and 121 vs. 114.

LC-ESI-MS/MS analysis was performed on an AB SCIEX LC-MS/MS (Triple TOF 5600 plus) system. Samples were subjected chromatography using a 90-min gradient consisting of 95–70% buffer A [0.1% (v/v) FA, and 5% (v/v) ACN] for 65 min, 30–50% buffer B [0.1% (v/v) FA, and 95% (v/v) ACN] for 5 min, 50–80% buffer B for 10 min, 80% buffer B for 5 min, and 80–5% buffer B for 5 min. Peptides were separated on a Magic C18 AQ column (5 μm,120 Å, 75 μm i.d. ×12 cm). MS1 spectra were collected from 350 to 1500 m/z for 250 ms. The 20 most intense precursors with charge states of 2–5 were selected for fragmentation, and MS2 spectra were collected in the range of 50–2000 m/z for 100 ms. Precursor ions were excluded from reselection for 15 s.

The MS/MS data were processed using the ProteinPilot v4.5 software for analysis. The Paragon algorithm, which is integrated into ProteinPilot, was employed for protein identification using the NCBI *Geobacter* database for database searching (Shilov et al., 2007). The peptide and protein false discovery rate (FDR) was estimated with an automatic decoy database search strategy using the PSPEP (Proteomics System Performance Evaluation Pipeline) algorithm. A two-tailed Student’s *t*-test was performed at a significance level of *P* ≤ 0.05.

Homology search of the identified protein sequences was performed with a localized Basic Local Alignment Search Tool for proteins (BLASTp) with default settings. Matches with the highest similarity scores and an *E*-value ≤ 1 × 10^-5^ were recorded for each protein. All identified protein sequences were also mapped into metabolic pathways using the Automatic Annotation server on Kyoto Encyclopedia of Genes and Genomes (KEGG). The subcellular localization prediction of proteins was performed using PSORTb version 3.0 (Yu et al., 2010).

## Results

### Extracellular Electron Transfer Capability of *G. soli*

**FIGURE 1:**
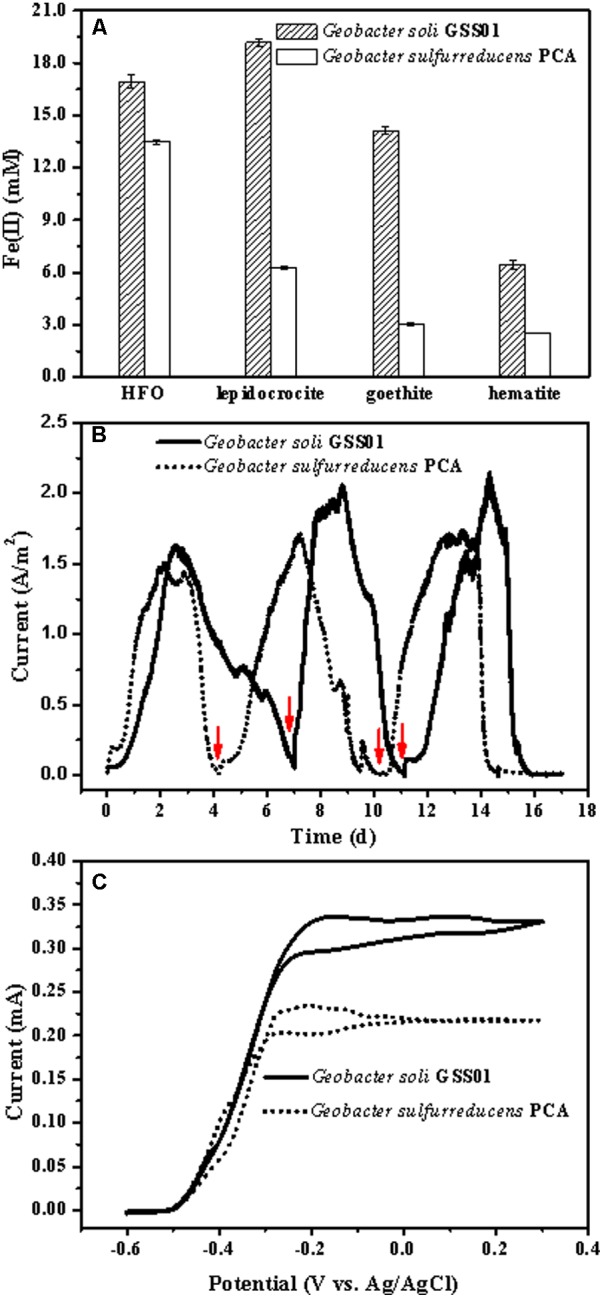
Comparison of the EET capability of *G. soli* GSS01 and *G. sulfurreducens* PCA. **(A)** Fe(III) oxide reduction following a 15-day reaction. **(B)** Electrical current generation. Arrows indicate the medium change. **(C)** CVs of electroactive bioanodes under turnover conditions (scan rate: 1 mV/s). Vertical bars indicate mean ± SD (standard deviation) of three replications. Results for electrical current generation and CVs are representative of triplicate biofilms.

As shown in **Figure 1A** and Supplementary Figure S2, both strains GSS01 and PCA were able to reduce four common insoluble Fe(III) oxides. After incubation for 15 days, the amount of extractable Fe(II) produced from FH, lepidocrocite, goethite, and hematite reduction by strain GSS01 was increased to 16.9 ± 0.4 mM, 19.2 ± 0.2 mM, 14.1 ± 0.2 mM, and 6.4 ± 0.2 mM, respectively, which was 1.3–4.7 times higher than that by strain PCA. After incubation for 30 days, both strains GSS01 and PCA reduced approximately 40% of the Fe(III) for FH, while strain GSS01 reduced substantially greater amounts of Fe(III) for hematite, goethite, and lepidocrocite (5.2–14.3% for strain PCA and 11.8–33.2% for strain GSS01). These results suggested that strain GSS01 might reduce Fe(III) oxides faster than strain PCA.

As shown in **Figure 1B**, after a short lag, the electrical current density generated by strains GSS01 and PCA exhibited a steep increase to a maximum value of 1.6 ± 0.1 A/m^2^ and 1.5 ± 0.1 A/m^2^, respectively. The peak electrical current density generated by strain GSS01 (2.2 ± 0.2 A/m^2^) was observed at the 3rd cycle, and was 30% higher than that generated by strain PCA (1.7 ± 0.1 A/m^2^). The Coulombic efficiency for the 2nd and 3rd cycles was calculated, yielding an average of 95.1 and 92.2% for strains GSS01 and PCA, respectively. As shown in **Figure 1C**, the catalytic electrical current of both strains was evaluated by CVs under turnover conditions, and the catalytic electrical current of strain GSS01 (0.34 ± 0.03 mA) was 1.5 times higher than that of strain PCA (0.23 ± 0.02 mA).

### Bioelectrochemical Characterization of *G. soli* Biofilms

**FIGURE 2:**
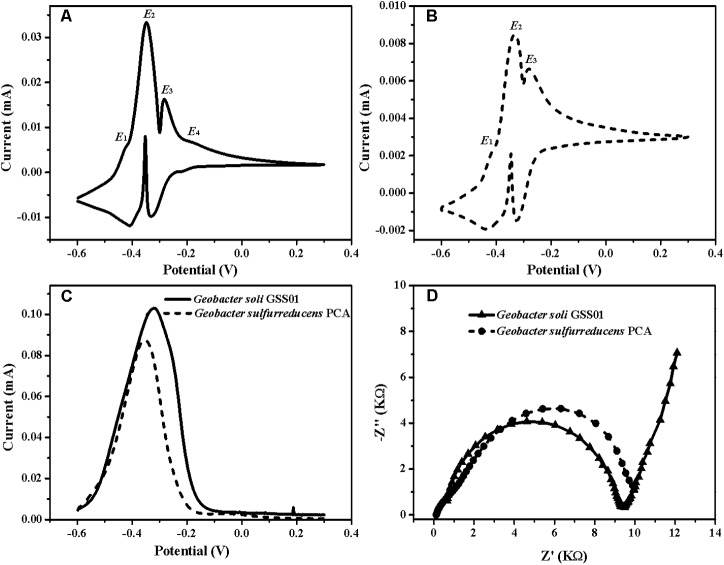
CVs of the anode biofilms of *G. soli* GSS01 **(A)** and *G. sulfurreducens* PCA **(B)** under the non-turnover conditions (scan rate: 1 mV/s). **(C)** DPV of the anode biofilms of *G. soli* GSS01 and *G. sulfurreducens* PCA under turnover conditions. **(D)** Nyquist plots of EIS spectra for the anode biofilms of *G. soli* GSS01 and *G. sulfurreducens* PCA. Results are representative of triplicate biofilms.

To accurately identify the biocatalytic active sites responsible for electricity generation, biofilms were analyzed by non-turnover CV and turnover DPV (Pous et al., 2016). As shown in **Figures 2A,B**, both biofilms demonstrated two major redox species with formal potentials of -0.38 V (*E*_2_) and -0.31 V (*E*_3_) and a minor redox species with a formal potential of -0.42 V (*E*_1_). In addition to these three redox species, another minor one with a formal potential of -0.20 V (*E*_4_) was present in *G. soli* biofilms (**Figure 2A**), which was confirmed by DPV that *G. soli* biofilms possessed high oxidative activity at potentials more positive than -0.20 V (**Figure 2C**). EIS analysis (**Figure 2D**) revealed that *G. soli* biofilms possessed lower charge transfer resistance (R_ct_) than that of *G. sulfurreducens*.

### Electrochemical *in Situ* FTIR Spectra Analysis of External Redox Proteins

**FIGURE 3:**
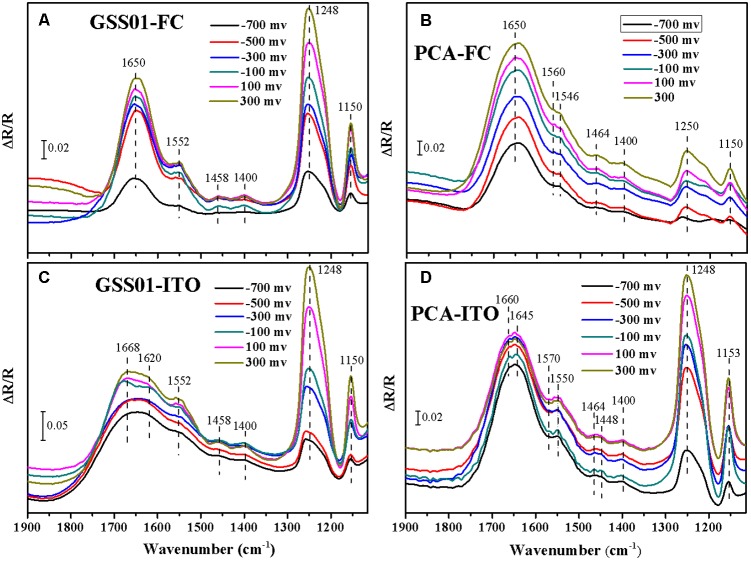
Electrochemical *in situ* FTIR spectra of *G. soli* GSS01 **(A,C)** and *G. sulfurreducens* PCA **(B,D)** grown with FC **(a,b)** and ITO **(C,D)**. Results are representative of three replicates.

Electrochemical *in situ* FTIR spectra were employed to obtain more information about the surface proteins of *G. soli* cells and monitor their conformational changes during electrochemical reactions at the molecular level. As shown in **Figures 3A,C**, positive bands appeared at 1552, 1458, 1400, 1248, and 1150 cm^-1^ for *G. soli* cells grown with FC and ITO. Compared to the spectra of cells grown with FC which had an obvious band at 1650 cm^-1^ (**Figure 3A**), the spectra of *G. soli* cells grown with ITO had one wider band at 1700–1600 cm^-1^ (**Figure 3C**). Furthermore, the band shoulder in **Figure 3C** moved from 1640 to 1670 cm^-1^ with a potential increased from -0.7 to 0.3 V, and the band was split into two bands at 1668 and 1620 cm^-1^ at -0.1 V.

The spectra of *G. sulfurreducens* cells grown with FC were dominated by a broad amide I band at ∼1650 cm^-1^ (**Figure 3B**). In addition to major bands at 1250 and 1150 cm^-1^, some minor bands were detected at 1560, 1546, 1464, and 1400 cm^-1^. The spectra for *G. sulfurreducens* cells grown with ITO were shown in **Figure 3D**; bands were detected at 1660, 1645, 1570, 1550, 1464, 1448, 1400, 1248, and 1153 cm^-1^. The amide I band in **Figure 3D** was split into two minor bands at 1660 and 1645 cm^-1^ at potentials -0.6 to 0.3 V; these differed from the spectra of *G. soli*, in which the amide I band was wider (1600–1700 cm^-1^) and was split only at -0.1 V (**Figure 3C**). Furthermore, there were three minor bands specific to *G. sulfurreducens* at 1570, 1464, and 1448 cm^-1^ and only one minor band at 1458 cm^-1^ for *G. soli*.

### Comparative Transcriptomic and Proteomic Analyses

A total of 2013 genes (961 upregulated and 1052 downregulated) showed significantly differential transcript levels (*P*-value ≤ 0.05) when *G. soli* cells grown with FH were compared with cells grown with FC. Comparing ITO-grown cells to FC-grown cells, a total of 1705 genes were differentially expressed at transcript level (*P*-value ≤ 0.05), of which 805 were increased and 900 were decreased. In the proteomic analysis, 2149 proteins were identified, of which 1928 (89.7%) proteins contained at least two unique peptides corresponding to approximately 60% of the predicted CDS of *G. soli* GSS01. Proteins that had differential abundance by more than 1.5-fold (*P*-value ≤ 0.05) in FH- or ITO-grown cells compared to FC-grown cells were summarized in Supplementary Table S1. One hundred and eighty-two and 35 proteins were upregulated in cells grown with FH and ITO, respectively; whereas, 227 and 172 proteins, respectively, were downregulated.

The number of differentially abundant proteins in proteomic analysis (409 for FH vs. FC and 207 for ITO vs. FC) was significantly less than that in transcriptomic analysis (2013 for FH vs. FC and 1705 for ITO vs. FC). In addition, many genes that had higher transcript levels in transcriptomic analysis, such as *cbcL*, *imcH*, *rplE*, and *rplN*, however, had reduced translation levels in proteomic analysis (Supplementary Tables S2, S3). This may be attributed to the low extraction yield for membrane-associated proteins or the post-transcriptional modulation of expression (Aklujkar et al., 2013).

#### Proteins Associated With Metabolism and Growth

**FIGURE 4:**
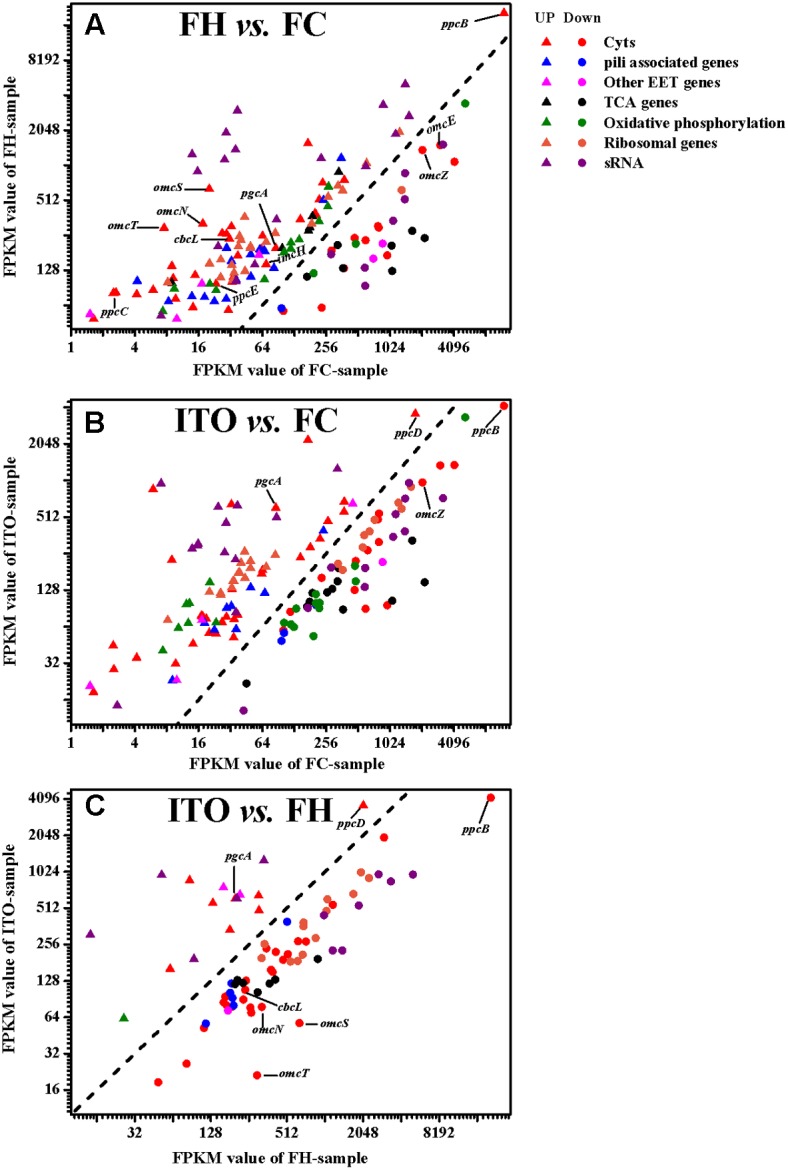
Comparison of transcript levels of selected genes that were differentially regulated in *G. soli* cells grown with FH vs. FC **(A)**, ITO vs. FC **(B)** and ITO vs. FH **(C)**. The genes in this figure are listed in Supplementary Table S2. The slope of the dashed line is 1.

**FIGURE 5:**
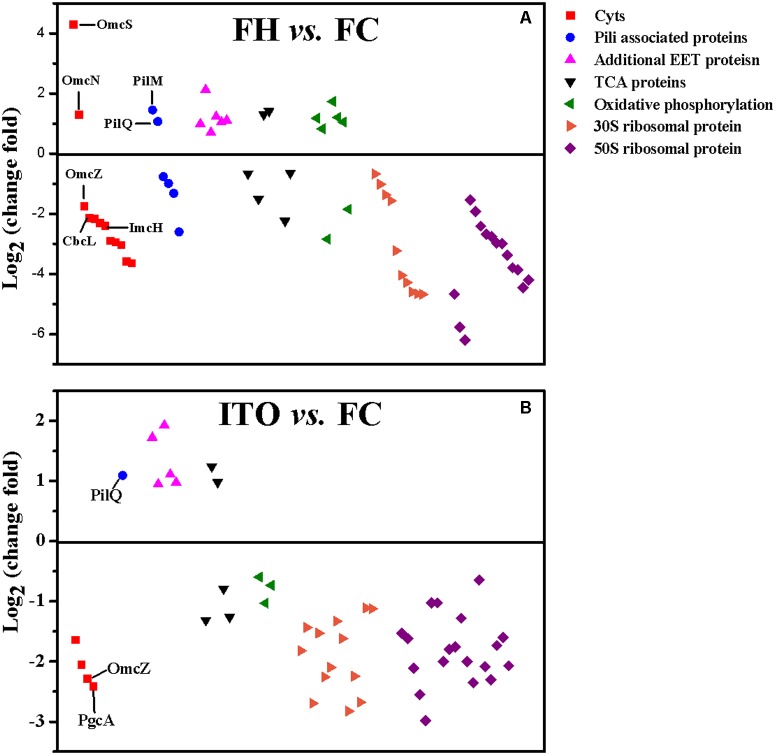
Comparison of the expression of selected proteins that had significantly differential abundance in *G. soli* cells grown with FH **(A)** and ITO **(B)** compared with FC. The proteins in this figure are listed in Supplementary Table S3.

Growth is usually slower with insoluble electron acceptors than with FC, and this can be reflected by the reduced abundance of many proteins involved in metabolism and translation (Lovley et al., 2004; Ding et al., 2008; Aklujkar et al., 2013). In this study, the expression levels of proteins associated with tricarboxylic acid (TCA) cycle, oxidative phosphorylation, and translation were compared in cells grown with FC, FH, and ITO (**Figures 4, 5** and Supplementary Tables S2, S3).

##### Tricarboxylic acid cycle

During growth with FH and ITO, the expression of citrate synthase (GltA), which is directly correlated with metabolic rates (Yun et al., 2011), was downregulated in both transcriptomic and proteomic analyses. In the transcriptomic analysis, the expressions of all three succinate dehydrogenase-encoding genes (*frdA*, *frdB* and *frdC*) were downregulated in cells grown with FH and ITO, as were the malate dehydrogenase (*mdh*)- and isocitrate dehydrogenase (*icd*)-encoding genes. This result was consistent with the slower rate of metabolism during growth with an insoluble electron acceptor in the previous studies. In addition, the transcript levels of many genes in TCA cycle, such as *frdB* and aconitate hydrotase-encoding gene *acnA*, had a less abundance in cells grown with ITO than with FH, indicating the lower metabolism rate in cells grown with ITO.

##### Oxidative phosphorylation

During growth with FH or ITO, the regulation of enzymes that catalyze the reactions of respiratory metabolism was complex. For example, in the proteomic analysis, one subunit of F_0_F_1_-type ATP synthase(AtpH) was more abundant in cells grown with FH than with FC, whereas another subunit (AtpG) was less abundant; in the transcriptomic analysis, the expression of cytochrome *c* oxidase (CoxA, CoxC and CoxD) was upregulated and the expression of cytochrome *bd* menaquinol oxidase (CydA) was downregulated. Similar to previous study of *G. sulfurreducens*, the two NADH dehydrogenase complexes were expressed under all tested conditions but the abundances of different subunits were mixed (Ding et al., 2008). Generally, most of the subunits had more abundance during growth with FH than with FC but had lower abundance during growth with ITO than with FC, which also indicates the lower metabolism rate in cells grown with ITO than with FH.

##### Ribosomal proteins

Greater ribosome production is associated with faster growth rates in many microorganisms (Franks et al., 2010). In the proteomic analysis, a number of ribosomal proteins had an approximately 1.5–25-fold lower abundance in cells grown with FH and ITO than with FC, which supported the conclusion that cell growth is slower with an insoluble electron acceptor (Lovley et al., 2004). However, the transcript levels of most of these ribosomal protein-encoding genes had a more abundance in cells grown with FH and ITO than with FC. This result indicated that the expression of ribosomal proteins was highly dependent upon post-transcriptional control as previously described (Mata et al., 2005).

#### Proteins Potentially Involved in EET

##### Cytochromes

In the transcriptomic analysis, a total of 62 *c*-Cyts-encoding genes had significantly differential transcript levels in cells grown with FH or ITO compared to with FC, among which, 23 *c*-Cyt-encoding genes showed higher transcript levels simultaneously in cells grown with FH and ITO (**Figure 4**, **Table 1**, and Supplementary Table S2). The *c*-Cyts-encoding gene that was increased most in transcript level in cells grown with FH vs. FC was SE37_02825 (*omcT*, 39.3-fold). Another *c*-Cyts-encoding gene SE37_02820 (*omcS*) which is predicted to be co-transcribed with gene SE37_02825 was increased by 31.8-fold in cells grown with FH vs. FC. The *c*-Cyts-encoding genes which were upregulated by more than 30-fold in FH-grown cells than in FC-grown cells included SE37_02870 (*pccJ*, 30.0-fold), SE37_01425 (*omcJ*, 32.5-fold), and SE37_02865 (30.9-fold). The transcript levels of *c*-Cyts-encoding genes that were increased most in cells grown with ITO vs. FC were SE37_03555 (25.3-fold), followed by SE37_11370 (*ccpA*, 19.9-fold) and SE37_01425 (*omcJ*, 17.9-fold). When comparing cells grown with ITO and FH, it was found that 27 *c*-Cyts-encoding genes, such as SE37_02820 (*omcS*), SE37_15520 (*cbcL*), and SE37_01725 (*omcB*), were downregulated in cells grown with ITO, and only 7 *c*-Cyts-encoding genes including SE37_05900 (*pgcA*) and SE37_11370 (*ccpA*) were upregulated.

**Table 1:** The selected important *c*-Cyts and additional proteins potentially involved in EET.

Locus ID	Gene name	Transcriptomics		Proteomics		Subcellular location
		FH vs. FC	ITO vs. FC	FH vs. FC	ITO vs. FC	
**Cytochromes**		
SE37_00350	*norC*	19.1^∗^	8.5^∗^	0.9	1.8	Cytoplasmic membrane
SE37_01425	*omcJ*	32.5^∗^	17.9^∗^	–	–	Unknown
SE37_01745	*omcB*	9.0^∗∗^	2.6^∗^	1.1	2.0	Unknown
SE37_02820	*omcS*	31.8^∗∗^	2.8^∗^	19.7^∗^	2.4	Periplasmic or extracellular
SE37_02825	*omcT*	39.3^∗∗^	2.8	2.2	1.0	Periplasmic
SE37_02835	–	10.0^∗∗^	2.6^∗^	–	–	Periplasmic
SE37_02865	–	30.9^∗∗^	9.9^∗^	–	–	Periplasmic or extracellular
SE37_02870	*pccJ*	30.0^∗∗^	11.3^∗∗^	–	–	Periplasmic
SE37_05900	*pgcA*	2.3^∗∗^	7.1^∗∗^	0.7	0.2^∗∗^	Extracellular
SE37_06935	–	4.3^∗∗^	3.3^∗∗^	0.6	2.4	Unknown
SE37_08180	–	3.1^∗∗^	3.9^∗∗^	0.9	1.0	Unknown
SE37_11360	*cccA*	9.2^∗∗^	12.8^∗∗^	0.5^∗^	0.6	Unknown
SE37_11370	*ccpA*	9.3^∗∗^	19.9^∗∗^	1.3	1.0	Periplasmic
SE37_11760	*omcN*	18.4^∗∗^	4.4^∗∗^	2.5^∗∗^	1.2	Periplasmic
SE37_11830	*omcO*	2.4^∗∗^	1.6^∗^	–	–	Unknown
SE37_11835	*omcP*	2.4^∗∗^	1.0	1.1	1.0	unknown
SE37_11920	*cbcR*	7.5^∗^	3.3^∗^	–	–	Unknown
SE37_00905	*omcE*	0.5^∗^	0.4^∗^	–	–	Outer membrane
SE37_04285	*omcZ*	–	0.5^∗^	0.3^∗^	0.2^∗^	Outer membrane
SE37_14295	–	11.9^∗^	5.2^∗^	0.4	0.5	Periplasmic
SE37_15310	–	7.9^∗^	3.8^∗^	1.0	1.2	Cytoplasmic membrane
SE37_13340	*imcH*	2.1^∗^	–	0.2^∗∗^	–	Cytoplasmic membrane
SE37_15520	*cbcL*	7.6^∗∗^	3.4^∗∗^	0.2^∗∗^	1.1	Cytoplasmic membrane
SE37_16115	*ppcB*	1.7^∗^	0.3^∗∗^	–	–	Periplasmic
SE37_16120	*ppcC*	31.9^∗∗^	11.3^∗∗^	–	–	Periplasmic
SE37_09795	*ppcD*	–	2.0^∗^	–	–	Periplasmic
SE37_05905	*ppcE*	4.2^∗∗^	2.4^∗^	–	–	Unknown
**Additional proteins associated with EET**		
SE37_04245	*hldE*	2.9^∗∗^	1.2^∗^	2.0^∗^	1.0	Cytoplasmic
SE37_08195	*ompB*	5.7^∗∗^	4.2^∗∗^	1.3	3.5^∗∗^	Unknown
SE37_12120	–	35.7^∗^	13.8	1.6^∗∗^	1.9^∗∗^	Outer membrane
SE37_11965	–	0.2^∗∗^	1.1^∗∗^	4.4^∗∗^	3.3^∗^	Outer membrane
SE37_10790	–	1.2^∗∗^	1.5^∗∗^	2.4^∗^	3.8^∗∗^	Outer membrane
SE37_11970	–	0.2^∗∗^	0.8^∗∗^	2.1^∗^	2.2^∗^	Extracellular
SE37_10395	–	4.9^∗∗^	2.3^∗^	2.2^∗∗^	2.0	Outer membrane

*Results are presented as the mean. –, not detected. ^∗^P-value ≤ 0.05, ^∗∗^P-value ≤0.01*.

Despite the higher transcript levels of many *c*-Cyts-encoding genes, only 2 *c*-Cyts (SE37_02820 and SE37_11760) were increased in cells grown with FH or ITO in the proteomic analysis (**Figure 5**, **Table 1**, and Supplementary Table S3). The expression of SE37_02820 was 19.7-fold higher in FH-grown cells than in FC-grown cells, and the expression of SE37_02820 was unchanged in ITO-grown cells compared to in FC-grown cells. SE37_11760, another *c*-Cyt, had a 2.5-fold more abundance in cells grown with FH than with FC, and unchanged in ITO-grown cells compared with FC-grown cells. In addition, 10 *c*-Cyts, such as SE37_04285 (OmcZ), SE37_15520 (CbcL), and SE37_13340 (ImcH), had a less abundance in cells grown with FH than with FC, and 4 *c*-Cyts, such as SE37_04285 (OmcZ) and SE37_05900 (PgcA), were downregulated in ITO-grown cells compared to FC-grown cells.

##### Pili associated proteins

*Geobacter soli* presumably forms electrically conductive pili that facilitate electron transfer to Fe(III) oxides (Holmes et al., 2016). In this study, genes predicted to encode pili-associated proteins in *G. soli* were detected in the transcriptomic analysis (Supplementary Figure S3). The *pilA* monomer gene (SE37_07695) demonstrated slight increases in transcript abundance in FH and ITO-grown cells (1.4- and 1.3-fold, respectively) (*P*-value = 0.11), although the changes for other genes associated with pili functions were somewhat higher. These results differed from those obtained for *G. sulfurreducens*, in which *pilA* had significantly higher transcript and translation levels in cells grown with FH vs. FC. According to the proteomic analysis, most pili-associated proteins were unchanged or downregulated, with the exception of PilQ (SE37_04515) and PilM (SE37_04495), which were upregulated by 2.1-fold and 2.7-fold in FH-grown cells, respectively. PilQ was also upregulated by 2.1-fold in ITO-grown cells. As previously reported, PilQ and PilM were required for pilus biogenesis and stability (Ayers et al., 2009).

##### Additional electron transfer proteins

SE37_08195, containing type I copper centers, was expressed at a level 3.5-fold higher in cells grown with ITO than in cells grown with FC according to proteomic analysis, and its transcript abundance was increased by 5.7- and 4.2-fold in cells grown with FH and ITO, respectively (**Table 1**). Another multicopper protein SE37_12120 was a ligand-gated channel in the outer membrane, and its transcript levels in cells grown with FH and ITO were 35.7 and 13.8 fold, respectively, higher than that in cells grown with FC. In the proteomic analysis, the abundance of SE37_12120 was increased by 1.6- and 2.0-fold in cells grown with FH and ITO, respectively.

SE37_11965 is an outer-membrane porin (OMP) protein that was expressed at levels 4.4- and 3.3-fold higher in cells grown with FH and ITO, respectively, in our proteomic analysis. The hypothetical protein SE37_11970, which is found in the same operon with SE37_11965, was translated at levels 2.1- and 2.2-fold higher in cells grown with FH and ITO, respectively, relative to cells grown with FC. Moreover, another OMP protein, SE37_10790, was expressed at levels 3.8-fold higher in cells grown with ITO. SE37_10395, an outer-membrane ligand-gated channel protein belonging to the porin superfamily, was increased by 4.9- and 2.2-fold in cells grown with FH in our transcriptomic and proteomic analyses, respectively.

##### Small Non-coding RNAs (sRNAs)

Comparative transcriptomics revealed that 30 novel sRNAs were expressed differentially in *G. soli* cells grown with insoluble electron acceptors vs. soluble acceptor, with 18 sRNAs increased and 8 sRNAs decreased in cells grown with FH, and 13 sRNAs increased and 12 sRNAs decreased in cells grown with ITO (**Table 2**). The most differentially expressed sRNA was sGS014, which was increased by up to 136.5-fold in cells grown with ITO. The expression levels of 18/30 (60%) sRNAs showed simultaneous change in cells grown with FH and ITO.

**Table 2:** Differentially expressed sRNA candidates.

sRNA_name	Location	Log_2_ (change fold)	
		FH vs. FC	ITO vs. FC
sGS015	925266–925409	0.7	-0.4
sGS016	976891–976971	-0.7	-1.0
sGS017	987247–987405	-1.7	-1.6
sGS038	1820786–1821265	-1.7	–
sGS052	2112941–2112874	-2.1	–2.1
sGS057	2302583–2302624	-	-1.7
sGS069	2492192–2492245	-0.7	-0.5
sGS077	2618504–2618318	-2.6	-1.6
sGS078	2603041–2603146	-1.4	-1.9
sGS092	2893738–2893775	-1.1	-2.1
sGS108	3387940–3388035	–	-0.9
sGS010	616821–616871	5.3	2.7
sGS014	904647–904537	2.9	7.1
sGS019	1172827–1173066	0.8	-0.7
sGS025	1363268–1363313	6.3	4.1
sGS026	1364430–1364574	5.3	3.2
sGS027	1365198–1365334	6.5	4.3
sGS029	1366154–1366217	5.9	4.2
sGS033	1625180–1625251	1.4	–
sGS037	1821003–1821156	–	2.0
sGS040	1832706–1832787	–	4.3
sGS041	1833954–1834063	3.1	4.7
sGS048	1986101–1986033	6.1	4.0
sGS050	2085347–2085392	1.8	–
sGS060	2339551–2339228	2.4	–
sGS080	2627960–2627828	3.1	2.4
sGS083	2740109–2739868	0.7	-1.1
sGS084	2775434–2775339	2.0	2.5
sGS097	2998494–2998370	1.5	1.2
sGS107	3384068–3384112	1.9	–

*The index of sRNA_name is ordered by the location of the sRNA. –, unchanged*.

To further investigate the potential role of sRNA in EET, the interaction between the differentially expressed sRNAs and the EET genes was analyzed using the RNA interaction prediction tool IntaRNA (Smith et al., 2010). As shown in Supplementary Table S4, the most increased sRNA sGS014 could interact with 30 EET genes, and two outer-membrane *c*-Cyts SE37_11760 and SE37_11830 were predicted to interact with up to 28 different sRNAs.

## Discussion

### *Geobacter soli* Is a Promising Exoelectrogen With Potential Distinctive EET Conduits

The electrical current generation by either strain GSS01 or PCA (2.2 A/m^2^ and 1.7 A/m^2^, respectively) herein was higher than that in previous reports (1.4 A/m^2^ and 1.1 A/m^2^, respectively) (Yang et al., 2017), and that is because the electrical current generation by the same strain differs when different electrode materials, electrode surfaces, or reactor structures are used (Wei et al., 2011; Zhu et al., 2014; Sun et al., 2015). According to previous reports, almost all members of the genus *Geobacter* are capable of generating electrical current, and the maximum electrical current generated may vary greatly from individual to individual, e.g., >2.6 A/m^2^ by *G. sulfurreducens* KN400, and <0.5 A/m^2^ by *G. chapellei* (Yi et al., 2009; Kato, 2017). In any event, strain GSS01 is a promising exoelectrogen which possesses the capacity for electrical current generation comparable to that of other well-known *Geobacter* members, such as *G. sulfurreducens* PCA and *G. anodireducens* SD-1 (approximately 2.1 A/m^2^) (Sun et al., 2015).

As previously reported, the voltammetric methods, including CV and DPV, can provide evidence for the interfacial redox proteins in the biofilms that directly interact with the electrode (Fricke et al., 2008). The results from CV and DPV revealed an extra redox protein present in *G. soli* biofilm, with a formal potential of -0.20 V (*E*_4_), compared to *G. sulfurreducens* biofilm. As outer-surface proteins with higher redox potential will facilitate the transfer of electrons outward from the periplasm, we speculated that the extra redox protein may contribute to the high EET capability of *G. soli* (Liu et al., 2011). The presence of the new extra redox protein in *G. soli* cell indicated that there might be an unknown electron transfer conduit in *G. soli*.

In the electrochemical *in situ* FTIR spectra, the intensities of the bands at 1248 and 1150 cm^-1^, which were previously reported in relation to *c*-Cyts (Marboutin et al., 2006; Busalmen et al., 2008), increased with the potential shift from -0.7 to 0.3 V. In addition, the band at 1400 cm^-1^ was related to vibrations of the heme ring (Busalmen et al., 2008). The assignment of the band at 1552 cm^-1^ was ambiguous because both amide II and heme modes absorb in this region (Ataka and Heberle, 2003). The bands appearing in the region between 1700 and 1600 cm^-1^ were all assigned to the amide I mode of the peptide backbone, which was primarily modulated by complex protein folding (Ataka and Heberle, 2004). The amide I-associated change with the potential shift indicated the presence of an enormous amount of outer-membrane redox proteins whose molecular conformation changed upon redox transition (Ataka and Heberle, 2004). The different vibrations of spectra shown in **Figures 3A,C** reflected the distinctive modes of the external proteins of *G. soli* cells caused by electron acceptors. Moreover, the significant differences of amide I band and other bands revealed in **Figures 3C,D** between *G. soli* and *G. sulfurreducens* reflected the contributions of distinctive external redox proteins to the electrochemical behaviors of the two *Geobacter* species, corroborating previous observations that the conduit of electrons across the periplasm and outer membrane is variable among *Geobacter* species (Butler et al., 2010; Smith et al., 2013).

### Proteins Potentially Involved in EET in *G. soli*

Current evidence from studies with *G. sulfurreducens* has revealed that many *c*-Cyts play an important role in EET: (1) inner-membrane electron transfer uses at least two different pathways, known as the CbcL- and ImcH-dependent pathways (Levar et al., 2014; Zacharoff et al., 2016); (2) electron transfer from inner- to outer-membrane might be mediated by PpcA family (PpcA-PpcE) (Butler et al., 2010; Morgado et al., 2010); (3) transferring electrons across the outer-membrane relies on trans-outer membrane porins (Liu et al., 2015); and (4) the terminal electron transfer from outer-membrane to electron acceptor is mediated by OmcS and OmcZ (Mehta et al., 2005; Nevin et al., 2009).

In the transcriptomic analysis, the expression of genes *cbcL* and *imcH* was upregulated by 7.6- and 2.1-fold in cells grown with FH than with FC, respectively, and unchanged in cells grown with ITO (**Table 1**). However, in the proteomic analysis, the protein CbcL had a less abundance in cells grown with FH than with FC. According to previous studies, there is at least two electron transfer pathways out of the *G. sulfurreducens* inner membrane quinone pool: the CbcL-dependent pathway operates at or below potentials of -0.1 V (vs. SHE), and the ImcH-dependent pathway operates only above this value (Levar et al., 2017). As the redox potentials of FC, FH, and ITO were different with each other, and the redox potentials of the cultures containing FC and FH will be reduced along with the Fe(III)-reduction (Levar et al., 2017), the regulation of these two proteins was directly dependent not only on the types of electron acceptors but also on the Fe(III)-reducing ratio in the cultures.

Among five closely related small triheme *c*-Cyts PpcA family, the transcript levels of *ppcB*, *ppcC*, and *ppcE* were upregulated in cells grown with FH than with FC, while the *ppcD* had a higher transcript lever in cells grown with ITO than with FC (**Figure 4** and Supplementary Table S2). These proteins are all predicted to have a periplasmic localization by PSORT (**Table 1**). Genetic studies have suggested that PpcA is an important component in FC reduction (Ding et al., 2008). It is also found that the redox potentials of PpcA-E are lower than outer-membrane *c*-Cyts such as OmcS and OmcZ, which is the opposite of the typical electron transfer chains designed (Santos et al., 2015).

OmcB, a dodecahaem *c*-Cyt embedded in the outer-membrane, is required for Fe(III)-reduction (Leang et al., 2003). The closest homologs to OmcB in *G. soli* are 10 or 12-heme *c*-Cyts, i.e., SE37_01745 and SE37_01725, with 84% and 65% amino acid sequence identity to OmcB, respectively. The *omcB* gene had higher transcript abundance in both FH- and ITO-grown cells (**Figure 4**), but its translation level was unchanged in the proteomic analysis (Supplementary Table S1). This result was consistent with previous proteomic study in *G. sulfurreducens* (Ding et al., 2008), which was supported by the finding that OmcB is important for the FC reduction as well as insoluble acceptors (Leang et al., 2003).

In the proteomic analysis, two increased *c*-Cyts in cells grown with FH or ITO were SE37_02820 and SE37_11760 (**Table 1**). The closest homolog to SE37_02820 in *G. sulfurreducens* is OmcS, with 94.7% amino acid sequence identity. Previous studies in *G. sulfurreducens* have verified that OmcS was the terminal Fe(III) oxides reductase but was not essential for electrode reduction (Mehta et al., 2005; Qian et al., 2011). SE37_11760 has 24 heme-biding sites and is predicted to be located in the periplasmic space. It shows 98.8% amino acid identity to OmcN in *G. sulfurreducens* which is reported to have less abundance in cells grown with insoluble electron acceptors and has been certified not essential for EET in *G. sulfurreducens* (Aklujkar et al., 2013).

OmcZ is found to play an important role in promoting electron transfer from the biofilm to the electrode surface but not to insoluble Fe(III) oxides (Nevin et al., 2009), and is specifically localized at the biofilm–anode interface in biofilms achieving high current density (Inoue et al., 2011). The transcript level of *omcZ* has been reported to be increased by more than 40 folds in electrode-grown cells compared to soluble electron acceptor-grown cells (Nevin et al., 2009); however, in present study, *omcZ* (SE37_04285) transcription and translation were decreased significantly in FH- and ITO-grown cells compared to FC-grown cells (**Table 1**).

PgcA is an extracellular *c*-Cyt acting as an electron shuttle, which can promote electron transfer from the cell surface to Fe(III) oxides but not electrodes (Zacharoff et al., 2017). SE37_05900 in *G. soli* is a homolog of PgcA, with an amino acid sequence similarity of 87.9%, and is predicted to be located in the extracellular matrix according to PSORTb. As its localization is the extracellular fraction, the proteomics of whole cells cannot reflect the variation of SE37_05900. In the transcriptomic analysis, it was upregulated significantly by 2.3- and 7.1-fold in cells grown with FH and ITO than FC, respectively. Based on current available evidence, it was uncertain if SE37_05900 was also used as electron shuttle in *G. soli*.

Multicopper protein SE37_08195 is a homolog of OmpB in *G. sulfurreducens*, which is required for the reduction of insoluble Fe(III) oxides (Mehta et al., 2006). This protein is predicted to differ from multicopper proteins found in other microorganisms because it contains sequences indicative of an Fe(III)-binding motif and a fibronectin type III domain (Holmes et al., 2008). Another multicopper protein SE37_12120 belongs to a family of proteobacterial TonB-dependent outer membrane receptor/transporters which bind and translocate copper ions, responsible for providing copper for the copper-containing proteins (Yoneyama and Nakae, 1996). The homolog of SE37_12120 in *G. sulfurreducens* is GSU2982, whose function has not previously been investigated. Unlike SE12120, GSU2982 has been reported to be downregulated in *G. sulfurreducens* cells grown with FH (Ding et al., 2008).

Porin-like proteins are often required for the proper processing and transport of outer-membrane proteins, including *c*-Cyts (Afkar et al., 2005). The significantly higher expression of four porin proteins, i.e., SE37_11965, SE37_11970, SE37_10790, and SE37_10395, indicated that they may play important roles in EET, although indirectly. In addition, SE37_10395 protein also belongs to the heme/hemoglobin receptor family, which is associated with the binding and utilization of hemoglobin and hemin (Noinaj et al., 2010). The higher expression of SE37_10395 may result from the demand for heme by cytochromes or other iron-containing proteins essential for EET. However, the role of this type of protein in heme transport and its potential function in EET are not yet clear in the genus *Geobacter*.

### Distinctive Regulation of the EET Proteins Might Be Involved in *Geobacter* Species

Transcriptomic and proteomic analyses have previously been performed to identify important genes for EET in *G. sulfurreducens* (Holmes et al., 2006; Ding et al., 2008; Nevin et al., 2009; Aklujkar et al., 2013; Kavanagh et al., 2016). Here, we compared important genes (including previously reported genes or those identified in this study) for EET in *G. soli* and *G. sulfurreducens* at both transcript and translation levels. In the proteomic analysis, OmcS was the only upregulated protein in both *G. soli* and *G. sulfurreducens* cells grown with FH vs. FC, which highlighted the importance of OmcS for the reduction of FH. However, a number of other *c*-Cyts and PilA proteins that were upregulated in *G. sulfurreducens* were unchanged in *G. soli* (Supplementary Figure S4a). Comparative proteomics of *G. sulfurreducens* indicated that proteins associated with multiple secretion systems, such as PulQ and PulE, are particularly important for electron-conducting biofilms vs. planktonic cells (Supplementary Figure S4b) (Kavanagh et al., 2016). However, these proteins, with the exception of the Type IV pilus secretion lipoprotein PilQ, were unchanged in *G. soli*. Importantly, the OMP proteins upregulated in *G. soli* were decreased or unchanged in *G. sulfurreducens*.

Large differences between *G. soli* and *G. sulfurreducens* were also observed in the transcriptomic analysis. For example, transcript levels of *omcE* and *pilA*, which are increased in *G. sulfurreducens* cells grown with FH, had lower abundances in *G. soli* cells grown with FH, and *omcJ* and *ppcC* transcripts that were increased by more than 30-fold in *G. soli* cells grown with FH are reported to be unchanged in *G. sulfurreducens* cells (Fig. S5a). For cells grown with electrodes, upregulated genes in *G. sulfurreducens*, such as *cbcA*, *omcE*, and *hybS*, were downregulated in *G. soli*, while the obviously upregulated genes *cccA*, *omcN*, *ppcC*, and *ppcE* in *G. soli* are reported to be unchanged in *G. sulfurreducens* (Supplementary Figure S5b). According to Smith et al. (2013), the proteins involved in EET in *G. sulfurreducens* and *G. metallireducens* are different, which is primarily attributed to the non-conserved genes in these two species. However, all the genes that are included in Supplementary Figures S4, S5 have homologs in *G. sulfurreducens* and *G. soli* (Supplementary Table S5), and therefore, the differential expression pattern of proteins in *G. sulfurreducens* and *G. soli* might result from their distinctive regulation at the transcript and translation levels.

sRNAs, ranging approximately from 40 to 500 nt in length, regulate gene expression at many levels, including RNA editing, RNA stability, translation, and post-translation (Gaimster et al., 2016). Available evidence suggests that many outer-membrane proteins in bacteria are regulated via sRNAs (Guillier et al., 2006). As outer-membrane proteins are important for EET, the differential expression of sRNAs was subsequently important. Qiu et al. (2010) have reported that several sRNAs showed differential expression in *G. sulfurreducens* under growth conditions using fumarate or FC as electron acceptor or under nitrogen fixing conditions. The present study found several sRNAs that have not been reported previously in the *Geobacter* genus, and analyzed the interaction between sRNAs and EET genes in the *Geobacter* genus for the first time (**Table 2** and Supplementary Table S4). As almost all the sRNAs identified are encoded in the intergenic regions which are under lower selection pressure (Nawaz et al., 2017), they have more room to mutate in different *Geobacter* species. The sRNAs may participate in the modulation of the expression of EET genes in different *Geobacter* species, and further efforts are required to determine their impact on species-specific modulation of gene expression.

In summary, this study has demonstrated that *G. soli* is a promising Fe(III) oxide reducer and electrical current producer, and its EET mechanisms are worthy of further research. The identification of several proteins which have significantly differential abundance in *G. soli* cells grown with insoluble electron acceptors vs. soluble electron acceptor generates a manageable list for future studies on the mechanisms for EET in this species. The possibility of sRNAs in modulating EET gene expression in the *Geobacter* genus provides a new insight to explore the EET mechanisms. Our future work will be focused on the development of a genetic system and antibodies in *G. soli*, as it will make it possible to determine the accurate role and localization of specific proteins.

## Author Contributions

XC and GY designed the experimental protocol and carried out the sample collection and data analysis. GY wrote a draft of the paper. LH and ZY carried out the electrochemical analysis. JW and SZ contributed to the critical review of the paper. All authors read, commented on, and approved the manuscript.

## Conflict of Interest Statement

The authors declare that the research was conducted in the absence of any commercial or financial relationships that could be construed as a potential conflict of interest.
