# Corrigendum: Transcriptomic, Proteomic, and Bioelectrochemical Characterization of an Exoelectrogen *Geobacter soli* Grown With Different Electron Acceptors

**DOI:** 10.3389/fmicb.2018.03111

**Published:** 2018-12-12

**Authors:** Xixi Cai, Lingyan Huang, Guiqin Yang, Zhen Yu, Junlin Wen, Shungui Zhou

**Affiliations:** ^1^Fujian Provincial Key Laboratory of Soil Environmental Health and Regulation, College of Resources and Environment, Fujian Agriculture and Forestry University, Fuzhou, China; ^2^Guangdong Key Laboratory of Integrated Agro-environmental Pollution Control and Management, Guangdong Institute of Eco-environmental Science and Technology, Guangzhou, China

**Keywords:** comparative transcriptomics, comparative proteomics, bioelectrochemistry, *Geobacter soli*, extracellular electron transfer

In the original article, there was an error. Phenol, benzoate, and benzaldehyde were incorrectly described as hydrocarbons.

A correction has been made to the **Introduction**, paragraph four:

“*Geobacter soli* GSS01, one of few *Geobacter* species isolated from soil, has many environmentally significant physiological properties that are not found in *G. sulfurreducens*, such as the ability to anaerobically oxidize aromatic compounds including phenol, benzoate, and benzaldehyde (Zhou et al., 2014).”

The authors apologize for this error and state that this does not change the scientific conclusions of the article in any way. The original article has been updated.
