# Supplementary material for: Transcriptomic, Proteomic, and Bioelectrochemical Characterization of an Exoelectrogen Geobacter soli Grown With Different Electron Acceptors
Source: Front Microbiol. 2018 Jun 15;9:1075. doi: 10.3389/fmicb.2018.01075 (PMC6013743; doi:10.3389/fmicb.2018.01075)
Supplement: Supplementary file 2 [file Table_3.pdf]

Supporting Information

Transcriptomics, proteomics and bioelectrochemical characterization of an exoelectrogen *Geobacter soli* grown with different electron acceptors

Xixi Cai[1], Lingyan Huang[1], Guiqin Yang[1,*], Zhen Yu[2], Junlin Wen[2], Shungui Zhou[1]

[1]Fujian Provincial Key Laboratory of Soil Environmental Health and Regulation, College of Resources and Environment, Fujian Agriculture and Forestry University, Fuzhou, China;
[2]Guangdong Key Laboratory of Integrated Agro-environmental Pollution Control and Management, Guangdong Institute of Eco-environmental Science & Technology, Guangzhou, China

Running Head: Extracellular electron transfer in *Geobater*

***Corresponding author:**
Guiqin Yang
E-mail: ygqhappy@163.com

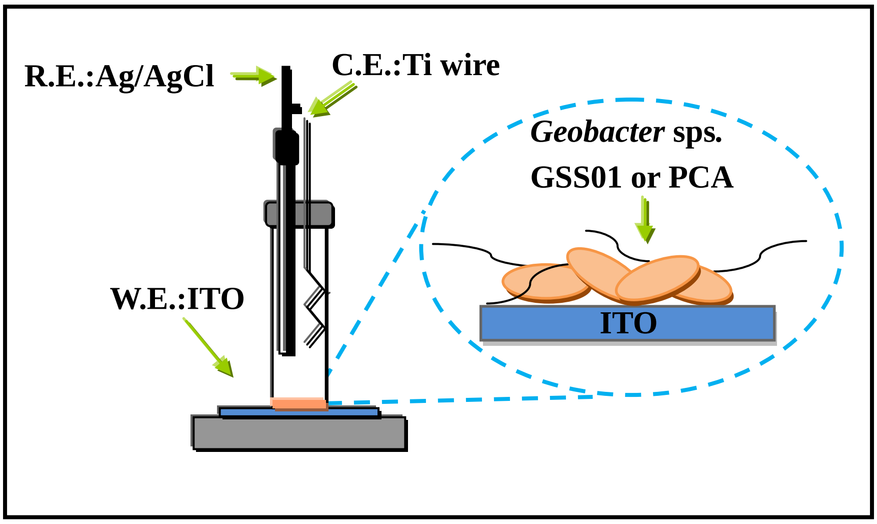

Fig. S1 Diagram of a single-chamber, three-electrode system using ITO as working electrode (W.E: ITO), Ag/AgCl electrode as the reference electrode (R.E: Ag/AgCl) and Ti wire as the counter electrode.

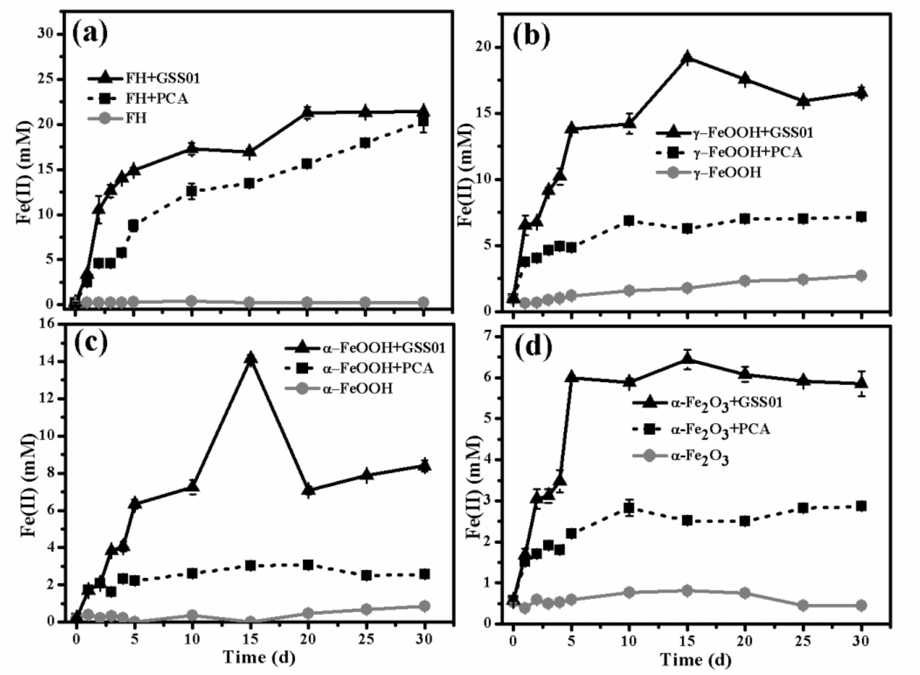

Fig. S2 Total Fe(II) production from the reduction of 50 mM ferrihydrite (HFO), lepidocrocite (γ-FeOOH), goethite (α-FeOOH) and hematite (α-Fe_2O_3) by strain GSS01 and PCA.

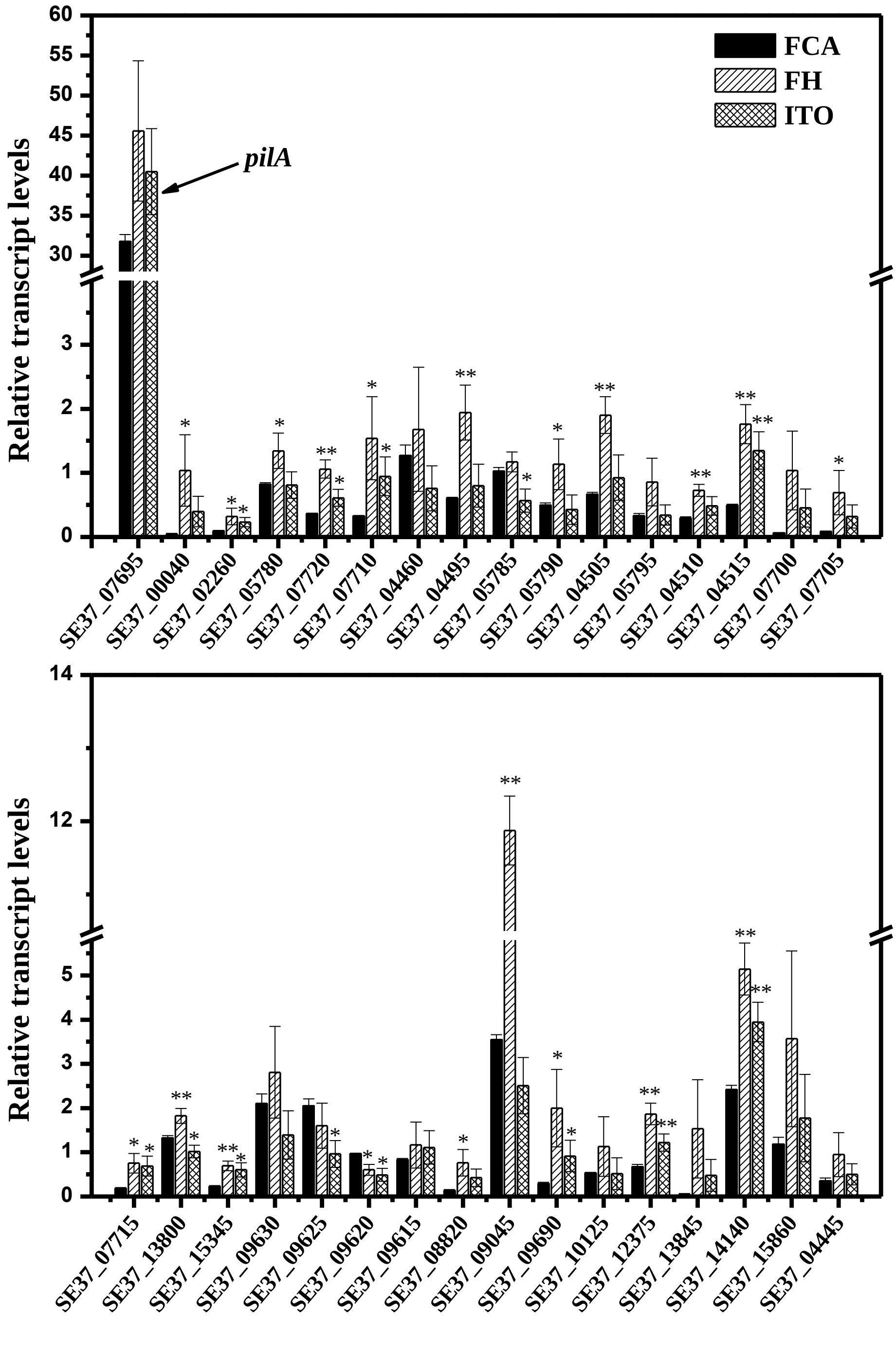

Fig. S3 Relative transcript levels of pilus-associated genes in *G. soli* cells grown with FH or electrode vs. FC.*, P<0.05; **, P<0.01

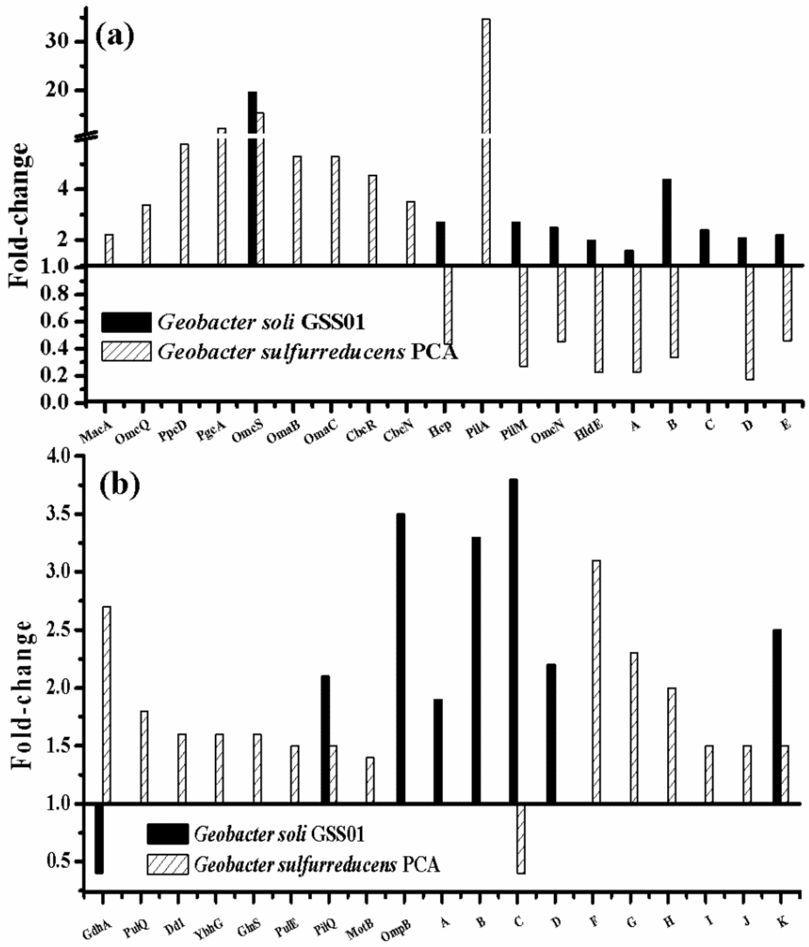

Fig. S4 Fold-change comparison of important proteins for electron transfer to Fe(III) oxides (a) or electrode (b) in *G. soli* and *G. sulfurreducens*. The data for *G. sulfurreducens* were obtained from Ding et al. (2008) and Kavanagh et al (2016). The y value was set as 1 for proteins that were not detected or were not significantly changed ($P \geq 0.05$) in the proteomics analysis. Proteins that have no name were labeled with capital letters. The annotation of proteins in this figure is available in Table S5.

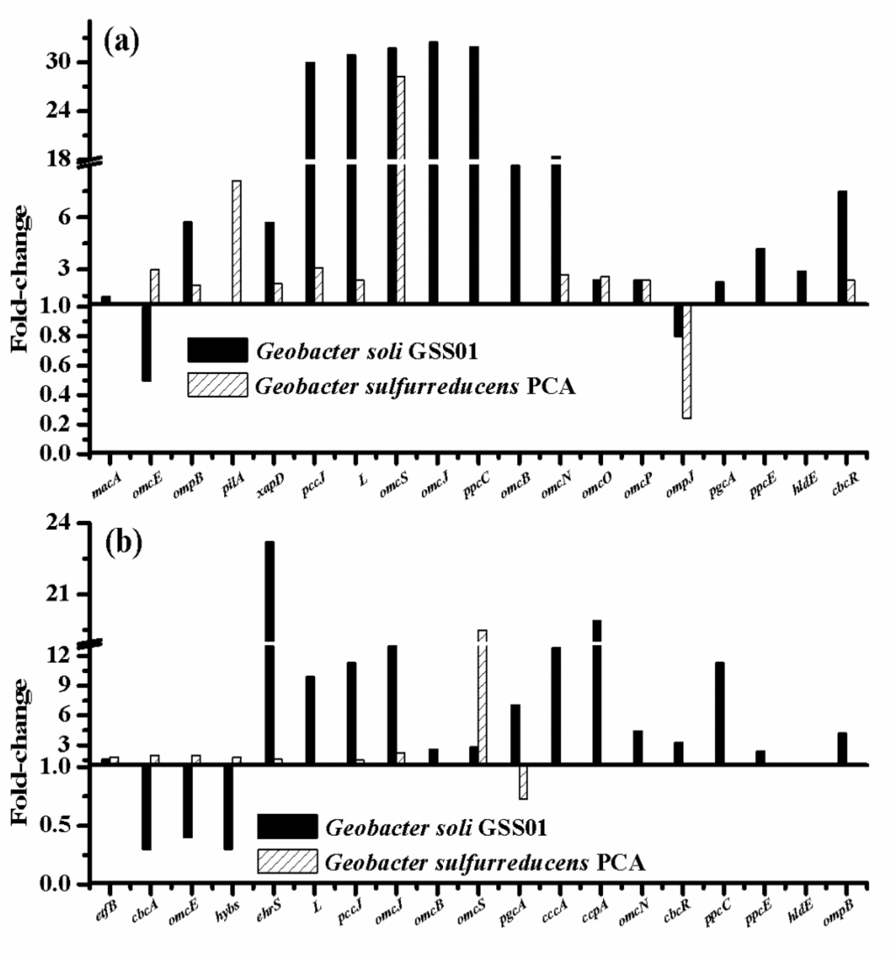

Fig. S5 Comparison of transcript changes of important genes for electron transfer to Fe(III) oxides (a) or electrode (b) in *G. soli and G. sulfurreducens*. The data for *G. sulfurreducens* were obtained from Aklujkar et al. (2013) and Holmes et al (2006).The y value was set as 1 for genes that were not detected or were not significantly changed ($P \geq 0.05$) in the transcriptomics analysis. Genes that have no name were labeled with capital letters. The annotation of genes in this figure is available in Table S5.

Table S2 Selected genes with significantly differential abundance (*P*≤0.05) in cells grown with FH or ITO in the transcriptomics analysis. -, unchanged or not find the gene names.

**Locus ID**	**Gene name**	**Annotation**	**Difference (change fold)**		
			**FH vs. FC**	**ITO vs. FC**	**ITO vs. FH**
**Proteins involved in metabolism and growth**					
**Tricarboxylic acid (TCA) cycle**					
SE37_02035	*aceF*	branched-chain alpha-keto acid dehydrogenase E2 subunit	11.2	-	-
SE37_07830	*korB*	2-oxoglutarate ferredoxin oxidoreductase subunit beta	1.7	0.6	0.3
SE37_03100	*pdhB*	pyruvate dehydrogenase E1 component subunit beta	1.6	-	-
SE37_10580	*acnA*	aconitate hydratase	2.0	-	0.6
SE37_14820	*pckA*	phosphoenolpyruvate carboxykinase	2.7	0.6	0.2
SE37_07825	*korC*	2-oxoglutarate:ferredoxin oxidoreductase subunit gamma	2.0	0.6	0.3
SE37_07845	*mdh*	malate dehydrogenase	0.6	0.5	-
SE37_09430	*gltA*	type I citrate synthase	0.2	0.2	-
SE37_07850	*icd*	isocitrate dehydrogenase	0.4	0.2	-
SE37_03070	*acn*	aconitate hydratase	0.7	0.6	-
SE37_09095	*frdB*	succinate dehydrogenase/fumarate reductase iron-sulfur subunit	0.2	0.1	0.6
SE37_09105	*frdC*	succinate dehydrogenase/fumarate reductase, cytochrome b558 subunit	0.1	0.1	-
SE37_09100	*frdA*	succinate dehydrogenase flavoprotein subunit	0.1	0.1	-
SE37_07835	*korA*	2-oxoglutarate ferredoxin oxidoreductase subunit alpha	-	0.5	0.3
SE37_07840	*korD*	2-oxoglutarate:ferredoxin oxidoreductase, ferredoxin subunit	-	0.5	0.5
SE37_06870	*acnB*	aconitate hydratase B	-	0.5	-
**Oxidative phosphorylation**					
SE37_14360	*buoF-2*	NADH dehydrogenase I subunit F	-	-	0.5
SE37_14370	*nuoE-1*	NADH dehydrogenase I subunit E	1.6	-	0.5
SE37_14375	*nuoBCD*	trifunctional NADH dehydrogenase I subunit B/C/D	-	0.7	-
SE37_14305	*nuoM-2*	NADH dehydrogenase I subunit M	7.8	5.5	-
SE37_13975	*atpA*	F0F1 ATP synthase subunit alpha	1.7	-	0.5
SE37_13985	*atpX*	ATP synthase F0, B' subunit	1.9	0.6	0.3
SE37_15965	*atpE*	ATP synthase F0 subunit C	1.7	-	-
SE37_13980	*atpH*	F0F1 ATP synthase subunit delta	1.8	0.7	0.4
SE37_16000	*nuoC*	NADH dehydrogenase I subunit C	-	0.5	-
SE37_16005	*nuoE*	NADH dehydrogenase I subunit D	-	0.5	-
SE37_16035	*nuoJ-1*	NADH dehydrogenase I subunit J	2.5	6.0	2.4
SE37_16055	*nuoN-1*	NADH dehydrogenase I subunit N	4.8	7.2	-
SE37_13990	*atpX*	ATP synthase F0, B' subunit	2.5	-	0.3
SE37_15305	*coxD*	cytochrome *c* oxidase, coo3-type, subunit IV	14.7	-	-
SE37_15300	*coxC*	cytochrome *c* oxidase, coo3-type, subunit III	9.4	-	-
SE37_15295	*coxA*	cytochrome *c* oxidase, coo3-type, cytochrome o subunit I	3.7	3.0	-
SE37_06970	*cydA*	cytochrome *bd* menaquinol oxidase, subunit I	0.7	0.6	
SE37_01760	*hoxE*	bidirectional hydrogenase complex protein HoxE	-	0.4	0.3
SE37_01770	*hoxU*	bidirectional hydrogenase complex protein HoxU	-	0.5	0.5
SE37_01765	*hoxF*	bidirectional NAD-reducing hydrogenase, diaphorase subunit	-	0.6	0.4
SE37_15970	*atpB*	ATP synthase F0 subunit A	-	-	0.5
SE37_14380	*nuoA-2*	NADH dehydrogenase I subunit A	0.4	0.3	-
SE37_15990	*nuoA-1*	NADH dehydrogenase I subunit A	-	0.4	-
SE37_15995	*nuoB*	NADH dehydrogenase I subunit B	0.6	0.3	0.4
SE37_16050	*nuoM-1*	NADH dehydrogenase I subunit M	-	7.6	-
SE37_16040	*nuoK-1*	NADH dehydrogenase I subunit K	-	5.3	-
SE37_16045	*nuoL-1*	NADH dehydrogenase I subunit L	-	8.0	-

**Ribosomal proteins**					
SE37_11530	*rpsC*	30S ribosomal protein S3	6.5	4.6	-
SE37_11500	*rplE*	50S ribosomal protein L5	8.5	6.1	-
SE37_11685	*rplM*	50S ribosomal protein L13	1.6	-	0.5
SE37_05020	*rpsB*	30S ribosomal protein S2	2.0	-	0.3
SE37_01155	*rpsR*	30S ribosomal protein S18	2.1	0.6	0.3
SE37_01150	*rpsF*	30S ribosomal protein S6	1.7	0.5	0.3
SE37_03850	*rpmB*	50S ribosomal protein L28	1.7	-	0.6
SE37_01135	*rplY*	50S ribosomal protein L25/general stress protein Ctc	1.7	-	0.6
SE37_11540	*rpsS*	30S ribosomal protein S19	3.2	2.9	-
SE37_11535	*rplV*	50S ribosomal protein L22	6.1	4.5	-
SE37_11525	*rolP*	50S ribosomal protein L16	4.3	4.1	-
SE37_11515	*rpsQ*	30S ribosomal protein S17	7.2	6.1	-
SE37_11510	*rplN*	50S ribosomal protein L14	5.8	5.4	-
SE37_11600	*rplL*	50S ribosomal protein L7/L12	3.2	2.9	-
SE37_11505	*rplX*	50S ribosomal protein L24	4.3	4.7	-
SE37_11495	*rpsH*	30S ribosomal protein S8	4.0	4.5	-
SE37_11490	*rplF*	50S ribosomal protein L6	5.4	4.7	-
SE37_11485	*rplR*	50S ribosomal protein L18	3.5	4.4	-
SE37_11480	*rpsE*	30S ribosomal protein S5	2.9	3.7	-
SE37_11475	*rpmD*	50S ribosomal protein L30	12.4	8.9	-
SE37_11470	*rplO*	50S ribosomal protein L15	3.1	4.5	-
SE37_11430	*rplQ*	50S ribosomal protein L17	4.2	4.0	-
SE37_12715	*rpmE*	50S ribosomal protein L31	0.5	0.5	-
SE37_11580	*rpsG*	30S ribosomal protein S7	-	0.6	0.5
SE37_13245	*rplU*	50S ribosomal protein L21	-	0.5	0.4
SE37_13240	*rpmA*	50S ribosomal protein L27	-	0.5	0.3
SE37_02290	*rpsA*	30S ribosomal protein S1	-	0.6	0.6
SE37_03985	*rpsT*	30S ribosomal protein S20	-	0.6	0.5
SE37_12645	*rpsU-1*	30S ribosomal protein S21	-	0.6	0.4
**Proteins involved in EET**					
**Cytochromes**					
SE37_00350	*norC*	cytochrome *c*	19.1	8.5	-
SE37_00895	-	cytochrome *c*	3.3	2.1	-
SE37_01700	*omcQ*	cytochrome *c*	2.0	1.8	-
SE37_02825	*omcT*	cytochrome *c*	39.3	-	0.1
SE37_02835	-	cytochrome *c*	10.0	2.6	0.3
SE37_02870	*pccJ*	cytochrome *c*	30.0	11.3	0.4
SE37_03555	-	cytochrome *c*	15.6	25.3	-
SE37_03965	-	cytochrome *c*	3.3	1.5	0.5
SE37_05900	*pgcA*	cytochrome *c*	2.3	7.1	3.1
SE37_05905	*ppcE*	cytochrome *c*	4.2	2.4	-
SE37_06935	-	cytochrome *c*	4.3	3.3	-
SE37_08180	-	cytochrome *c*	3.1	3.9	
SE37_08880	*omcI*	cytochrome *c*	4.6	2.1	0.5
SE37_11360	*cccA*	cytochrome *c*	9.2	12.8	-
SE37_11370	*ccpA*	cytochrome *c*	9.3	19.9	2.1
SE37_11705	-	cytochrome *c*	1.9	-	0.4
SE37_11760	*omcN*	cytochrome *c*	18.4	4.4	0.3
SE37_11765	-	cytochrome *c*	3.1	-	0.4
SE37_11830	*omcO*	cytochrome *c*	2.4	1.6	0.7
SE37_11835	*omcP*	cytochrome *c*	2.4	-	0.4
SE37_11905	-	cytochrome *c*	3.1	-	-
SE37_11920	*cbcR*	cytochrome *c*	7.5	3.3	-
SE37_11940	-	cytochrome *c*	1.9	2.8	-
SE37_11945	-	cytochrome *c*	2.0	-	0.4

SE37_13340	*imcH*	cytochrome *c*	2.1	-	-
SE37_14185	-	cytochrome *c*	3.9	2.9	-
SE37_14295	-	cytochrome *c*	11.9	-	-
SE37_15065	-	cytochrome *c*	3.1	-	0.4
SE37_15310	-	cytochrome *c*	7.9	-	-
SE37_15520	*cbcL*	cytochrome *c*	7.6	-	0.5
SE37_16115	*ppcB*	cytochrome *c*	1.7	0.3	0.2
SE37_16120	*ppcC*	cytochrome *c*	31.9	11.3	-
SE37_01425	*omcJ*	cytochrome *c*	32.5	17.9	-
SE37_02820	*omcS*	cytochrome *c*	31.8	2.8	0.1
SE37_02865	-	cytochrome *c*	30.9	-	0.3
SE37_01725	*omcB*	cytochrome *c*	9.0	2.6	0.3
SE37_00770	-	cytochrome *c*	0.7	-	-
SE37_00775	*obcC*	cytochrome *c*	0.4	0.4	-
SE37_00785	-	cytochrome *c*	0.3	0.3	-
SE37_00905	*omcE*	cytochrome *c*	0.5	0.4	-
SE37_01435	-	cytochrome *c*	0.3	0.7	2.7
SE37_02090	-	cytochrome *c*	0.4	0.1	0.4
SE37_02785	-	cytochrome *c*	0.2	0.1	0.6
SE37_05760	-	cytochrome *c*	0.6	0.6	-
SE37_08440	-	cytochrome *c*	-	-	0.5
SE37_00575	-	cytochrome *c*	-	-	0.6
SE37_00875	-	cytochrome *c*	-	-	0.7
SE37_09635	-	cytochrome *c*	-	-	0.4
SE37_11335	-	cytochrome *c*	0.4	0.6	1.6
SE37_11345	-	cytochrome *c*	0.5	0.3	0.5
SE37_11955	-	cytochrome *c*	0.4	1.5	4.3
SE37_00200	*macA*	cytochrome *c*	1.4	1.6	-
SE37_01170	*omcX*	cytochrome *c*	-	1.9	-
SE37_07370	-	cytochrome *c*	-	3.9	-
SE37_09795	*ppcD*	cytochrome *c*	-	2.0	1.8
SE37_12935	*nrfA*	cytochrome *c*	-	4.6	-
SE37_12940	*nrfH*	cytochrome *c*	-	2.8	-
SE37_16085	-	cytochrome *c*	-	1.5	1.9
SE37_04285	*omcZ*	cytochrome *c*	-	0.5	-
SE37_08660	-	cytochrome *c*	-	0.4	0.4
SE37_11710	-	cytochrome *c*	-	0.5	0.5
SE37_12855	-	cytochrome *c*	-	0.7	0.5
**Pili**					
SE37_00040	*pilB*	pilus assembly protein PilB	24.6	-	-
SE37_02260	*pilB*	pilus assembly protein PilB	3.5	2.6	-
SE37_05780	*pilB*	pilus assembly protein PilB	1.6	-	-
SE37_05785	*pilM*	pilus assembly protein PilM	-	0.6	0.5
SE37_07720	*pilB*	pilus assembly protein PilB	2.9	1.7	-
SE37_07710	*pilC*	pilus assembly protein PilC	4.7	2.9	-
SE37_04495	*pilM*	pilus assembly protein PilM	3.2	-	0.4
SE37_05790	*pilN*	pilus assembly protein PilN	2.3	-	-
SE37_04505	*pilO*	pilus assembly protein PilQ	2.9	-	0.5
SE37_04510	*pilP*	pilus assembly protein PilP	2.5	-	-
SE37_04515	*pilQ*	pilus assembly protein PilQ	3.5	2.7	-
SE37_07705	*pilS*	pilus assembly protein PilS	8.3	-	-
SE37_07715	*pilT*	pili twitching motility protein PilT	4.1	3.8	-
SE37_15345	*pilT*	pili twitching motility protein PilT	3.1	2.7	-
SE37_08820	*pilZ*	pilus assembly protein PilZ	5.5	-	-
SE37_09045	*pilZ*	pilus assembly protein PilZ	3.3	-	-
SE37_09690	*pilZ*	pilus assembly protein PilZ	6.8	3.1	-
SE37_12375	*pilZ*	pilus assembly protein PilZ	2.8	1.8	0.7

SE37_13800	*pilT*	pili twitching motility protein PilT	-	-	0.6
SE37_14140	*pilZ*	pilus assembly protein PilZ	2.1	1.6	0.8
SE37_09620	*pilX*	pilus assembly protein PilX	0.6	0.5	-
SE37_09625	*pilW*	pilus assembly protein PilW	-	0.5	-
**Other EET genes**					
SE37_04245	*hldE*	bifunctional heptose 7-phosphate kinase/heptose 1-phosphate adenyltransferase	2.9	-	0.4
SE37_08195	*ompB*	laccase	5.7	4.2	-
SE37_12120	-	ligand-gated channel	35.7	13.8	-
SE37_10395	-	ligand-gated channel	4.9	2.3	-
SE37_11970	-	hypothetical protein	0.2	-	3.0
SE37_11965	-	porin	0.2	-	4.7

Table S3 Selected proteins with significantly differential abundance ($P \leq 0.05$) in cells grown with FH or ITO in the proteomics analysis. -, unchanged or not find the gene names.

Locus ID	Gene name	Annotation	Difference (change fold)	
			FH vs. FC	ITO vs. FC
**Proteins involved in metabolism and cell growth**				
**TCA cycle**				
SE37_14820	*pckA*	phosphoenolpyruvate carboxykinase	0.6	-
SE37_09430	*gltA*	type I citrate synthase	0.4	0.4
SE37_10580	*acnA*	aconitate hydratase	2.5	2.4
SE37_11700	*icd*	3-isopropylmalate dehydrogenase	2.7	-
SE37_09945	*fumB*	fumarate hydratase	0.2	0.6
SE37_05525	*asl*	L-aspartate oxidase	0.6	-
SE37_06870	*acnB*	aconitate hydratase B	-	0.4
**Oxidative phosphorylation**				
SE37_15995	*nuoB*	NADH-quinone oxidoreductase subunit B	2.3	-
SE37_16005	*nuoE*	NADH dehydrogenase	1.8	-
SE37_16015	*nuoF-1*	NADH dehydrogenase	0.1	0.7
SE37_13965	*atpB*	ATP synthase subunit beta	3.3	-
SE37_13980	*atpH*	ATP synthase F0F1 subunit delta	2.3	-
SE37_13970	*atpG*	ATP F0F1 synthase subunit gamma	0.3	-
SE37_16020	*nuoG*	NADH dehydrogenase	-	0.5
SE37_00450	-	NADH-ubiquinone oxidoreductase subunit 3	-	0.6
**Ribosomal proteins**				
SE37_11440	*rpsD*	30S ribosomal protein S4	0.04	0.3
SE37_09005	*rpsA*	30S ribosomal protein S1	0.6	0.4
SE37_11495	*rpsH*	30S ribosomal protein S8	0.5	0.2
SE37_01150	*rpsF*	30S ribosomal protein S6	0.4	0.4
SE37_05020	*rpsB*	30S ribosomal protein S2	0.3	0.2
SE37_11680	*rpsI*	30S ribosomal protein S9	0.1	0.2
SE37_11530	*rpsC*	30S ribosomal protein S3	0.1	0.4
SE37_11445	*rpsK*	30S ribosomal protein S11	0.1	0.3
SE37_11450	*rpsM*	30S ribosomal protein S13	0.1	0.1
SE37_11540	*rpsS*	30S ribosomal protein S19	0.1	0.2
SE37_11685	*rplM*	50S ribosomal protein L13	0.1	0.2
SE37_11505	*rplX*	50S ribosomal protein L24	0.1	0.3
SE37_11635	*rpmG*	50S ribosomal protein L33	0.1	-
SE37_11605	*rplJ*	50S ribosomal protein L10	0.3	0.3
SE37_07210	*rplG*	50S ribosomal protein L7	0.3	-
SE37_11510	*rplN*	50S ribosomal protein L14	0.2	0.4
SE37_11610	*rplA*	50S ribosomal protein L1	0.2	0.2
SE37_11550	*rplW*	50S ribosomal protein L23	0.1	0.2
SE37_11470	*rplO*	50S ribosomal protein L15	0.1	0.6
SE37_11560	*rplC*	50S ribosomal protein L3	0.1	0.2
SE37_07150	*rpmF*	50S ribosomal protein L32	0.1	-
SE37_01165	*rpli*	50S ribosomal protein L9	0.1	0.2
SE37_11500	*rplE*	50S ribosomal protein L5	0.07	0.3
SE37_01040	*rplS*	50S ribosomal protein L19	0.05	0.3
SE37_11490	*rplF*	50S ribosomal protein L6	0.05	0.2
SE37_01015	*rpsP*	30S ribosomal protein S16	-	0.2
SE37_02290	*rpsA*	30S ribosomal protein S1	-	0.5
SE37_12645	*rpsU-1*	30S ribosomal protein S21	-	0.5
SE37_11520	*rpmC*	50S ribosomal protein L29	-	0.3
SE37_11615	*rplK*	50S ribosomal protein L11	-	0.3
SE37_13245	*rplU*	50S ribosomal protein L21	-	0.2

SE37_11555	*rplD*	50S ribosomal protein L4	-	0.2
SE37_01135	*rplY*	50S ribosomal protein L25	-	0.5
SE37_11600	*rplL*	50S ribosomal protein L7/L12	-	0.5
SE37_11685	*rplM*	50S ribosomal protein L13	-	0.2
SE37_11505	*rplX*	50S ribosomal protein L24	-	0.3
SE37_11605	*rplJ*	50S ribosomal protein L10	-	0.3
SE37_11510	*rplN*	50S ribosomal protein L14	-	0.4
SE37_11610	*rplA*	50S ribosomal protein L1	-	0.2
SE37_11550	*rplW*	50S ribosomal protein L23	-	0.2
SE37_11470	*rplO*	50S ribosomal protein L15	-	0.6
SE37_11560	*rplC*	50S ribosomal protein L3	-	0.2
SE37_01165	*rplI*	50S ribosomal protein L9	-	0.2
SE37_01040	*rplI*	50S ribosomal protein L19	-	0.3
SE37_11490	*rplF*	50S ribosomal protein L6	-	0.2
**Proteins involved in EET**				
**Cytchromes**				
SE37_02820	*omcS*	cytochrome *C*	19.7	-
SE37_11760	*omcN*	cytochrome *C*	2.5	-
SE37_04285	*omcZ*	cytochrome *C*	0.3	0.2
SE37_15520	*cbcL*	cytochrome *C*	0.2	-
SE37_00785	*nrfB*	cytochrome *C*	0.2	-
SE37_11705	-	cytochrome *C*	0.2	0.2
SE37_13340	*imcH*	cytochrome *C*	0.2	-
SE37_12935	*nrfA*	cytochrome *C*	0.1	-
SE37_04675	-	cytochrome *C*	0.1	-
SE37_08660	-	cytochrome *C*	0.1	-
SE37_01170	*omcX*	cytochrome *C*	0.1	-
SE37_01700	-	cytochrome *C*	0.1	-
SE37_05900	*pgcA*	cytochrome *C*	-	0.2
SE37_11710	-	cytochrome *C*	-	0.3
**Pili proteins**				
SE37_04495	*pilM*	pilus assembly protein PilM	2.7	-
SE37_04515	*pilQ*	pilus assembly protein PilQ	2.1	2.1
SE37_07715	*pilT*	type IV pili twitching motility protein PilT	0.6	-
SE37_02260	*pilB*	pilus assembly protein PilB	0.5	-
SE37_07710	*pilC*	pilus assembly protein PilC	0.4	-
SE37_04505	*pilO*	pilus assembly protein PilO	0.2	-
**Other EET proteins**				
SE37_04245	*hldE*	bifunctional heptose 7-phosphate kinase/heptose 1-phosphate adenyltransferase	2.0	-
SE37_11965	-	porin	4.4	3.3
SE37_08195	*ompB*	laccase		
SE37_12120	-	ligand-gated channel	1.6	1.9
SE37_10790	-	membrane protein	2.4	3.8
SE37_11970	-	hypothetical protein	2.1	2.2
SE37_10395	-	ligand-gated channel	2.2	2.0

Table S5 Annotation of genes in Fig. S4 and Fig. S5

Gene name	annotation	Locus ID in strain PCA	Locus ID in strain GSS01	Amino acid similarity
omcS	Cytochrome *c*	GSU2504	SE37_02820	94.7%
omcB	Cytochrome *c*	GSU2737	SE37_01745	83.8%
omcE	Cytochrome *c*	GSU0618	SE37_00905	93.5%
xapD	ABC transporter, ATP-binding protein	GSU1501	SE37_07675	59.5%
macA	Cytochrome *c* peroxidase	GSU0466	SE37_00200	95.7%
ompB	Laccase family multicopper oxidase	GSU1394	SE37_08195	94.9%
pccJ	Cytochrome *c*	GSU2494	SE37_02870	97.9%
omcJ	Cytochrome *c*	GSU0701	SE37_01425	98.1%
ppcC	Cytochrome *c*	GSU0365	SE37_16120	93.7%
ppcD	Cytochrome *c*	GSU1024	SE37_09795	97.8%
ppcE	Cytochrome *c*	GSU1760	SE37_05905	91.1%
pgcA	Lipoprotein cytochrome *c*	GSU1761	SE37_05900	88.1%
omcN	Lipoprotein cytochrome *c*	GSU2898	SE37_11760	98.8%
omcO	Cytochrome *c*	GSU2912	SE37_11830	98.5%
omcP	Cytochrome *c*	GSU2913	SE37_11835	97.1%
omcQ	Cytochrome *c*	GSU0592	SE37_00775	97.6%
ompJ	Outer membrane channel	GSU3304	SE37_13550	54.3%
ccdA	*Cytochrome c* biogenesis protein	GSU2085	SE37_04245	96.1%
cbcR	Methaquinol oxidoreductase complex, liproteotein cytochrome *c* subunit	GSU2930	SE37_11920	94.0%
hybS	Ni/Fe hydrogenase, small subunit	GSU0782	SE37_10925	98.7%
ehrS	NAD-dependent dehydrogenase subunit	GSU0745	SE37_01620	96.8%
cccA	Cytochrome B6	GSU2811	SE37_11360	95.9%
ccpA	Cytochrome *c* peroxidase	GSU2813	SE37_11370	98.0%
omaB	Cytochrome *c*	GSU2732	SE37_01740	97.4%
omaC	Cytochrome *c*	GSU2738	SE37_01720	97.4%
cbcN	Methaquinol oxidoreductase complex Cbc6, cytochrome *c* subunit	GSU2934	SE37_11940	98.1%
hcp	Iron-sulfur-oxygen hybrid cluster protein	GSU0674	SE37_01195	98.5%
PilA	Geopilin domain 1 protein	GSU1496	SE37_07695	85.3%
pilM	Type IV pilus biogenesis ATPase	GSU2032	SE37_04495	97.4%
pilQ	Type IV pilus secretin lipoprotein	GSU2028	SE37_04515	87.2%
gdhA	Glutamate dehydrogenase	GSU1305	SE37_08570	98.4%
pulQ	Type II secretion system secretin lipoprotein	GSU1778	SE37_05805	92.5%
pulE	Type II secretion system ATPase	GSU1783	SE37_05780	96.9%
ddl	D-alanine-D-alanine ligase	GSU3066	SE37_12520	95.6%
ybhG	Efflux pump, RND family, membrane fusion protein	GSU2823	SE37_11390	95.7%
glnS	Glutamine-tRNA ligase	GSU3366	SE37_14915	98.4%
motB	Peptidoglycan-binding lipoprotein, OmpA family	GSU1013	SE37_09840	96.9%
A	Ligand-gated channel, TonB-dependent copper receptor	GSU2982	SE37_12120	96.7%
B	Outer-membrane porin protein	GSU2939	SE37_11965	94.8%
C	Outer-membrane channels, porin superfamily	GSU0810	SE37_10790	92.1%
D	Hypothetical protein	GSU2940	SE37_11970	69.6%
E	Ligand-gated channel, porin superfamily	GSU0883	SE37_10395	82.5%
F	Glutamate synthase, FMN-Fe(II)-binding domain protein	GSU1239	SE37_08825	99.8%
G	FAD-dependent pyridine nucleotide-disulfide oxidoreductase	GSU1237	SE37_08835	97.5%
H	Efflux pump, RND family, membrane protein	GSU0496	SE37_00385	98.8%
I	Transcriptional regulator, MarR family	GSU2362	SE37_03395	93.0%
J	Phosphoglucomutase/phosphomannomutase family protein	GSU2013	SE37_04590	96.6%
K	Amino acid aminotransferase	GSU0117	SE37_13945	96.1%
L	Cytochrome *c*	GSU2495	SE37_02865	97.4%

References

Aklujkar, M., Coppi, M.V., Leang, C., Kim, B.C., Chavan, M.A., Perpetua, L.A., Giloteaux, L., Liu, A., and Holmes, D.E. (2013). Proteins involved in electron transfer to Fe(III) and Mn (IV) oxides by *Geobacter sulfurreducens* and *Geobacter uraniireducens*. *Microbiology* 159, 515-535.

Ding, Y.R., Hixson, K.K., Aklujkar, M.A., Lipton, M.S., Smith, R.D., Lovley, D.R., and Mester, T. (2008). Proteome of *Geobacter sulfurreducens* grown with Fe(III) oxide or Fe(III) citrate as the electron acceptor. *Biochim. Biophys. Acta* 1784, 1935-1941.

Holmes, D.E., Chaudhuri, S.K., Nevin, K.P., Mehta, T., Methé, B.A., Liu, A., Ward, J.E., Woodard, T.L., Webster, J., and Lovley, D.R. (2006). Microarray and genetic analysis of electron transfer to electrodes in *Geobacter sulfurreducens*. *Environ. Microbiol.* 8, 1805-1815.

Kavanagh, P., Botting, C.H., Jana, P.S., Leech, D., and Abram, F. (2016). Comparative proteomics implicates a role for multiple secretion systems in electrode-respiring *Geobacter sulfurreducens* biofilms. *J. Proteome Res.* 15, 4135-4145.
